# Supplementary material for: Identification of two short peptide motifs from serine/arginine-rich protein ribonucleic acid recognition motif-1 domain acting as splicing regulators
Source: PeerJ. 2023 Sep 18;11:e16103. doi: 10.7717/peerj.16103 (PMC10512959; doi:10.7717/peerj.16103)

Fig1 B

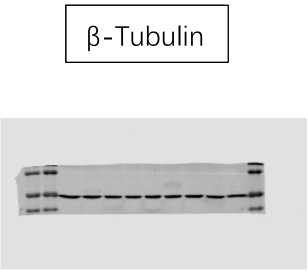

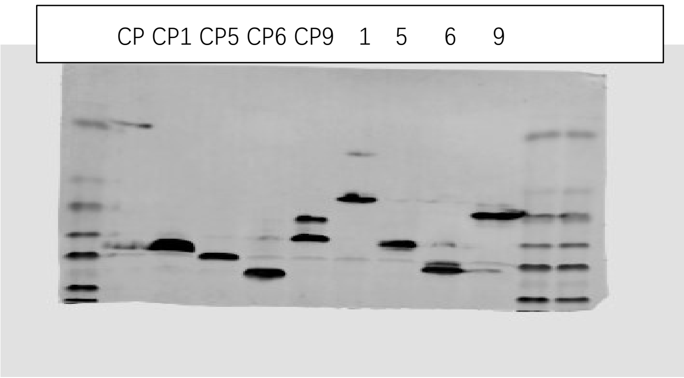

Fig1 C

Fig1 D

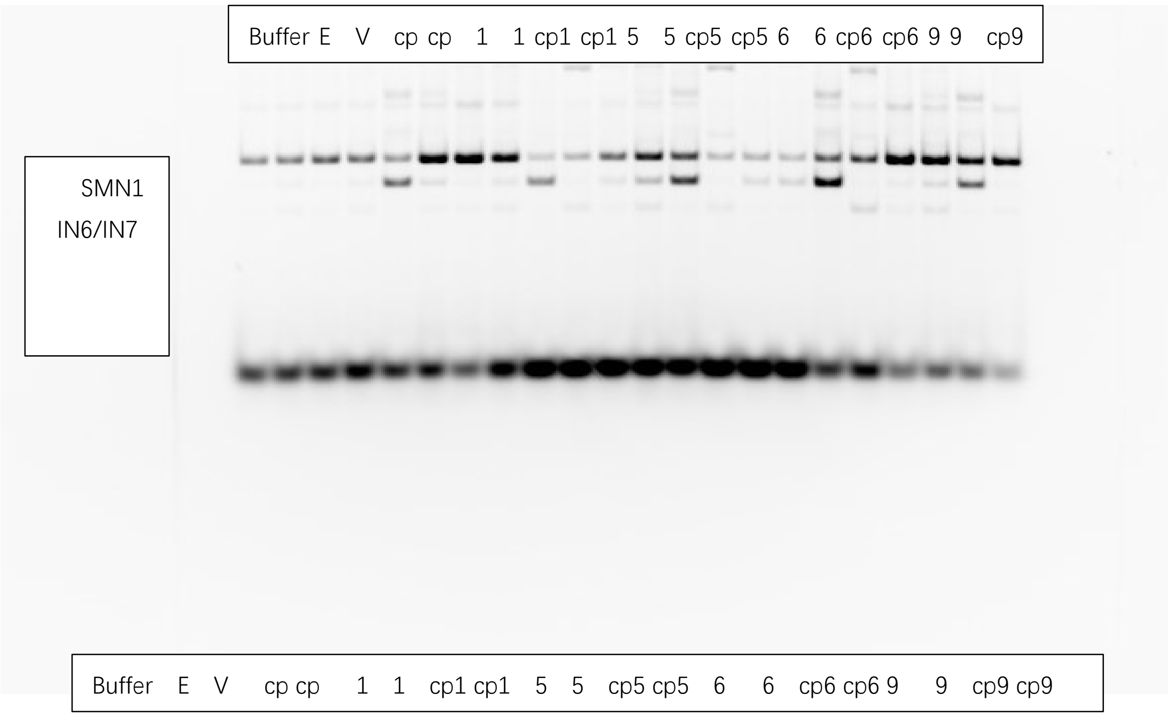

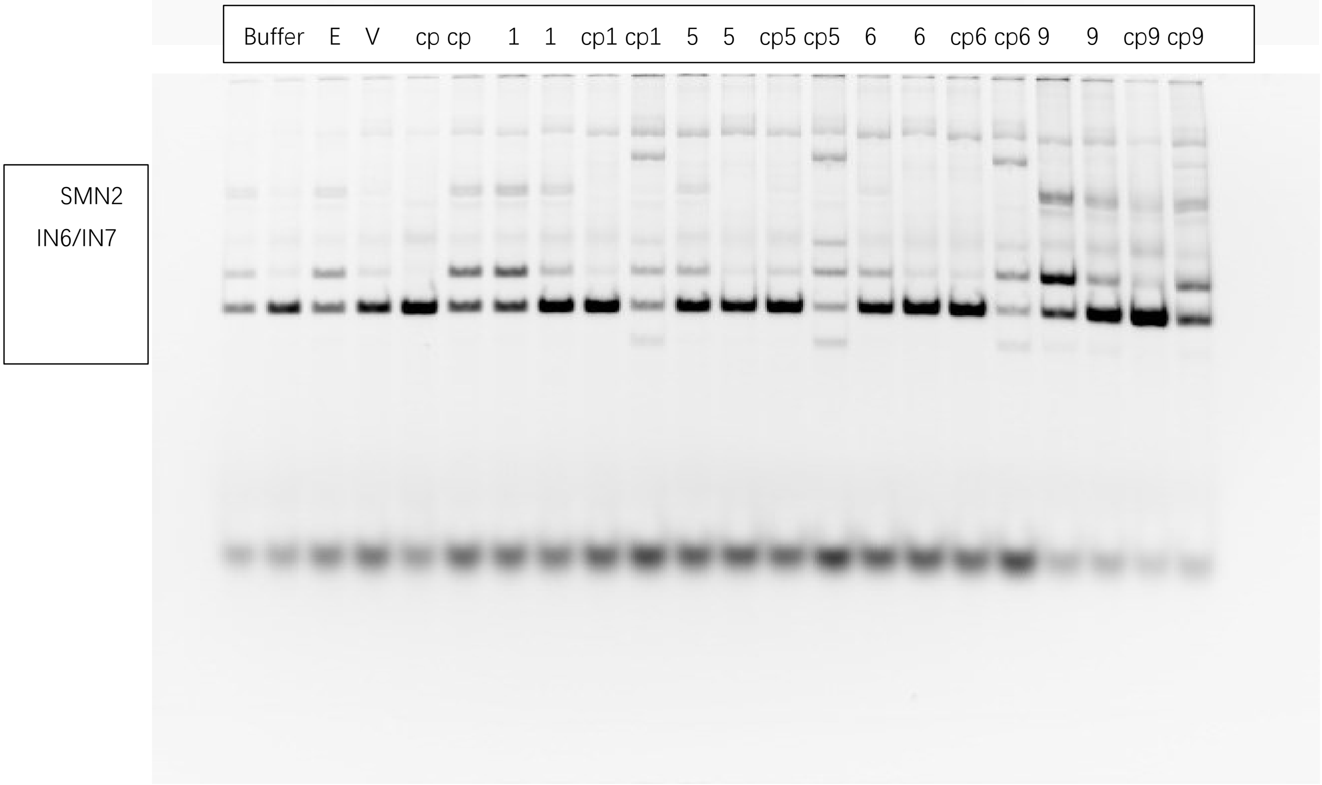

Fig1 B

2/RS △1 Gly/2 △RS 1/Gly RS 2 1 CP1 CP

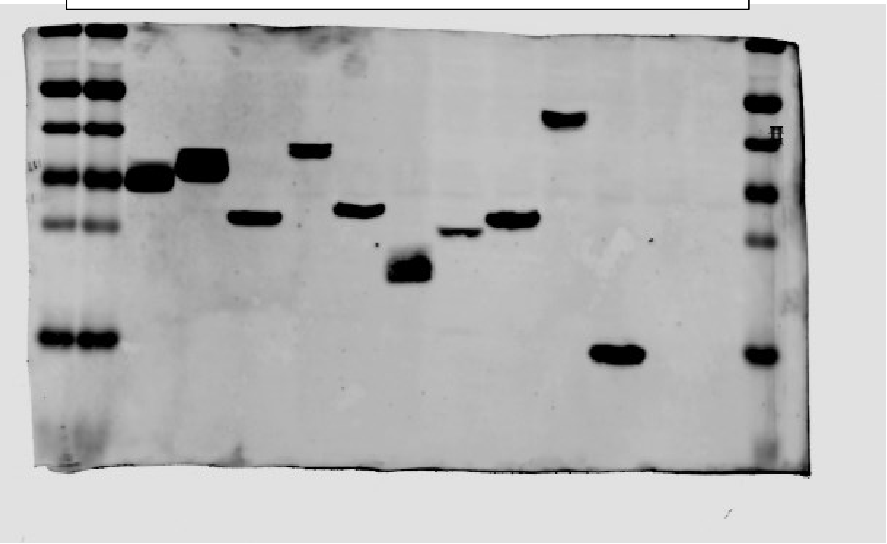

GAPDH

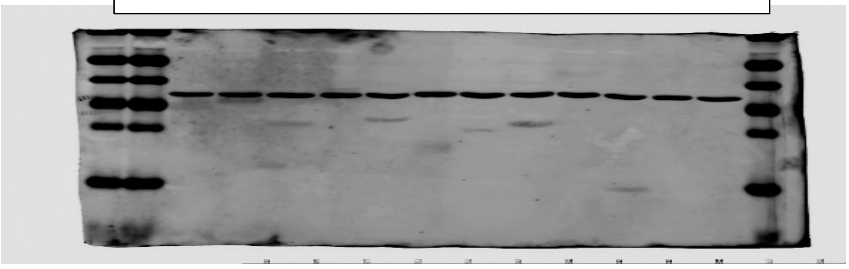

Fig2 C

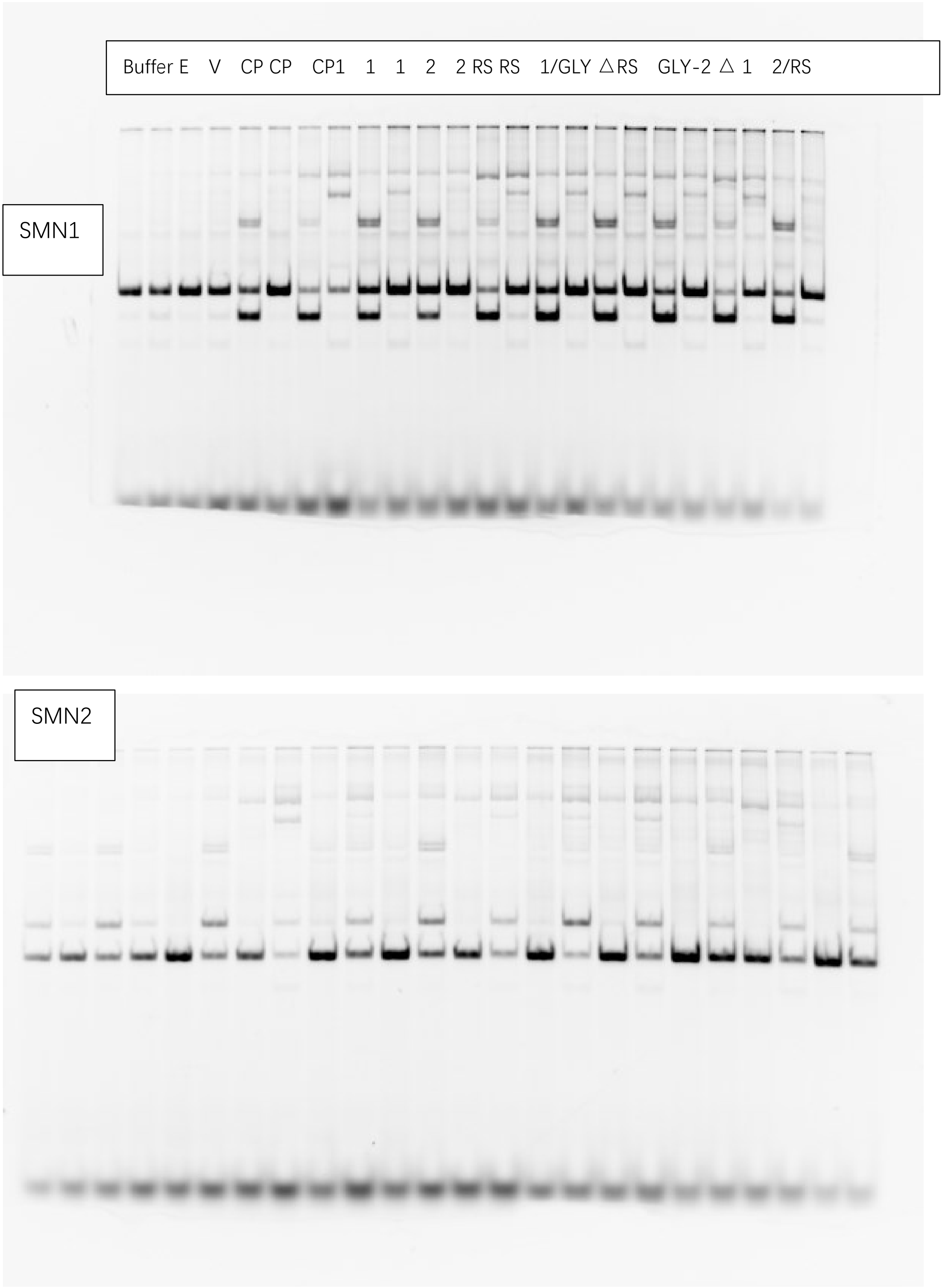

Fig2 D

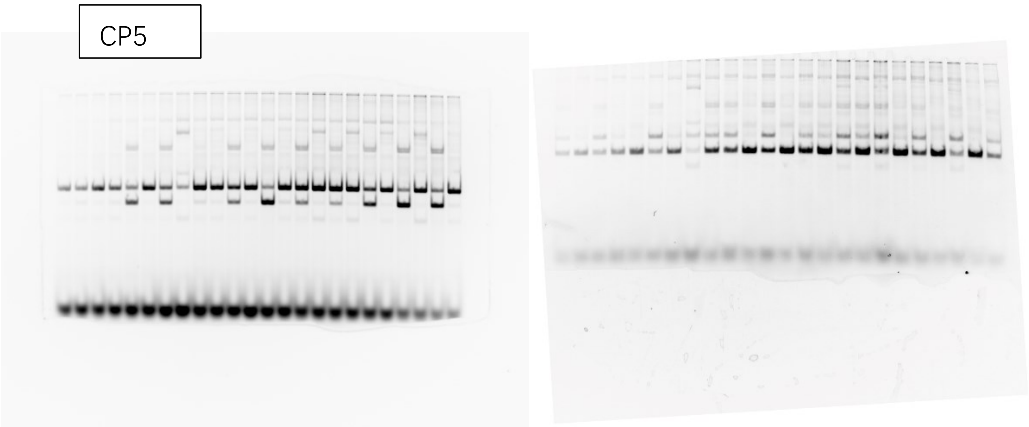

Fig2 E

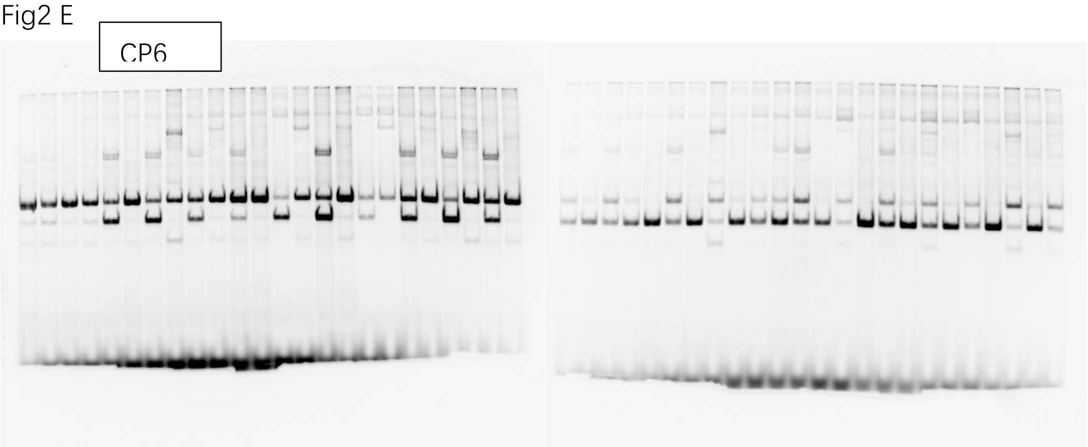

Fig2 F

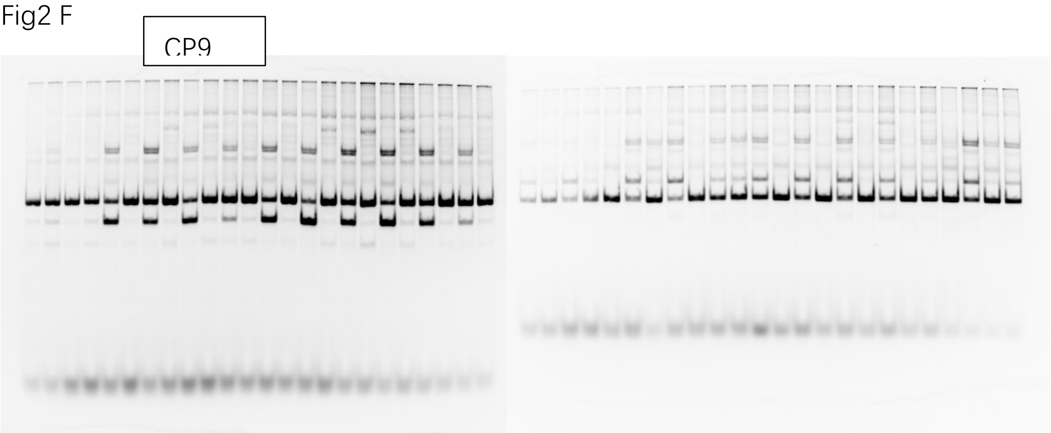

Fig3 B

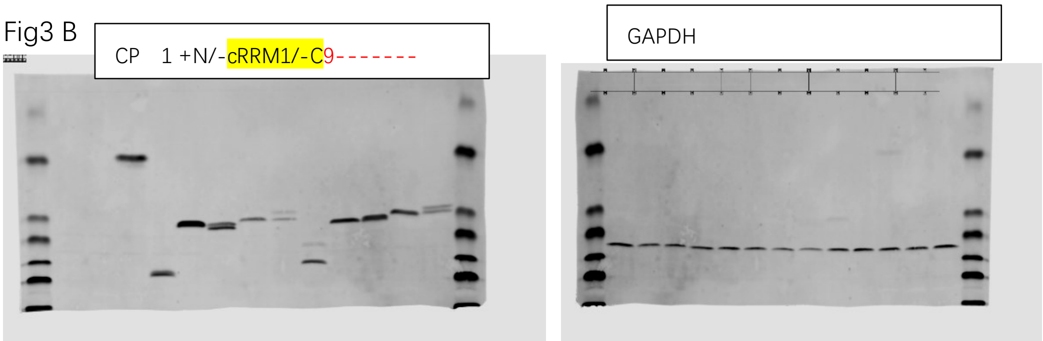

Fig3 C/D

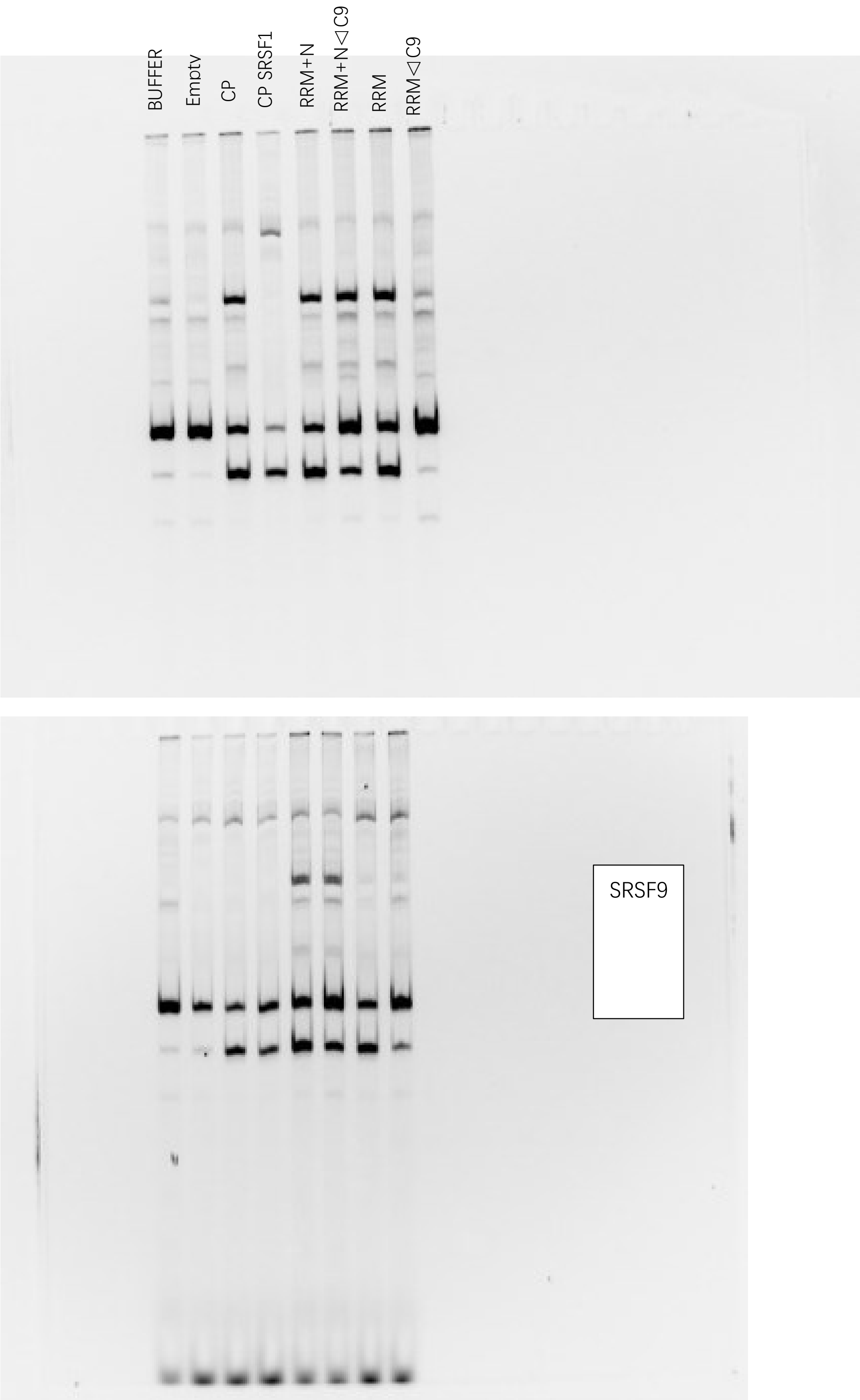

Fig3 E

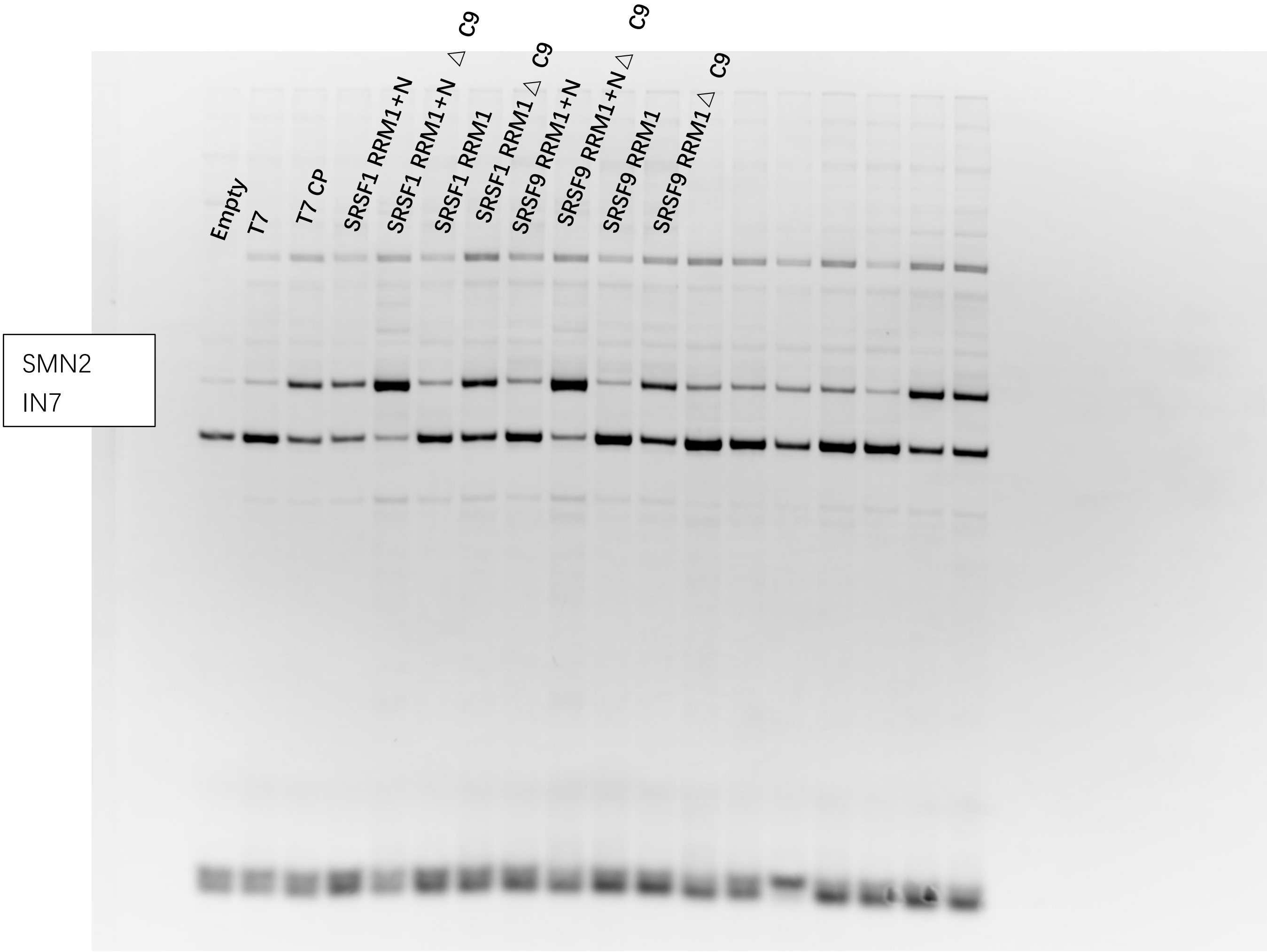
Empty
T7
T7 CP
SRSF1 RRM1+N
SRSF1 RRM1+N △ C9
SRSF1 RRM1
SRSF1 RRM1△ C9
SRSF9 RRM1+N
SRSF9 RRM1+N △ C9
SRSF9 RRM1
SRSF9 RRM1△ C9
SMN2
IN7

Fig3 F

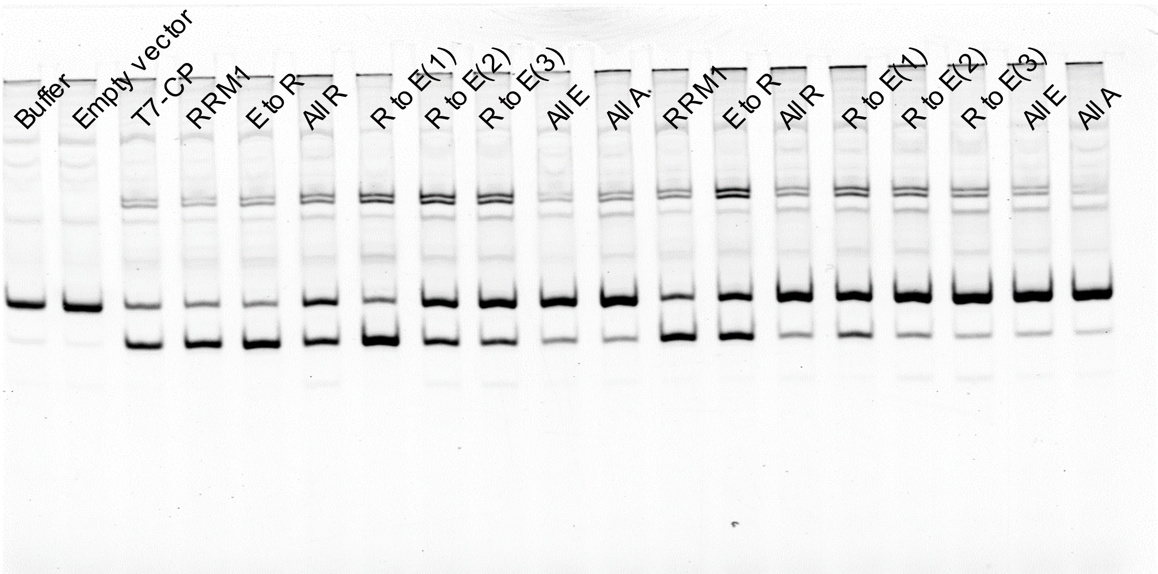
Buffer
Empty vector
T7-CP
RRM1
E to R
All R
R to E(1)
R to E(2)
R to E(3)
All E
All A
RRM1
E to R
All R
R to E(1)
R to E(2)
R to E(3)
All E
All A

Fig4 B （Left）

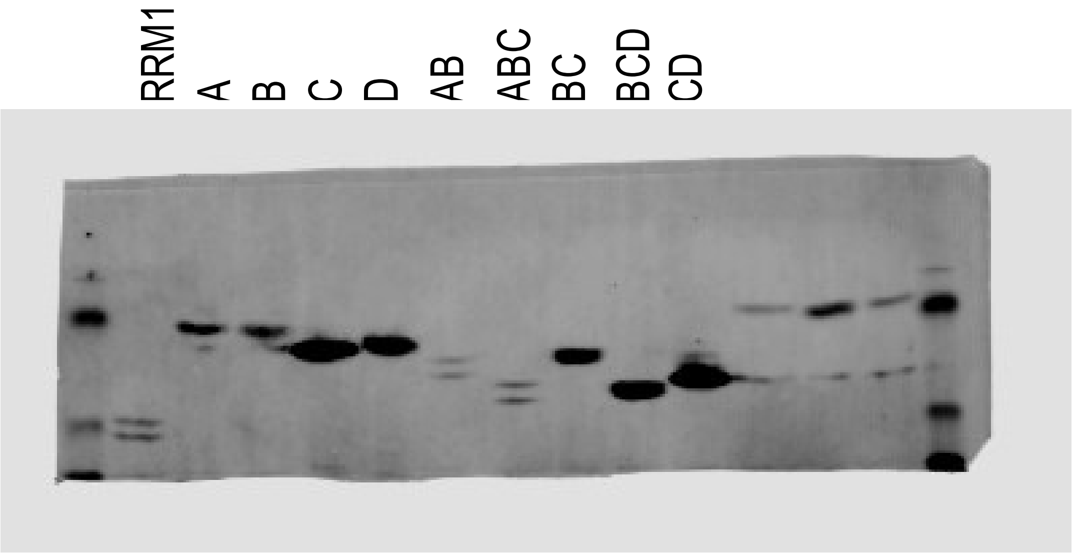

β-Tubulin

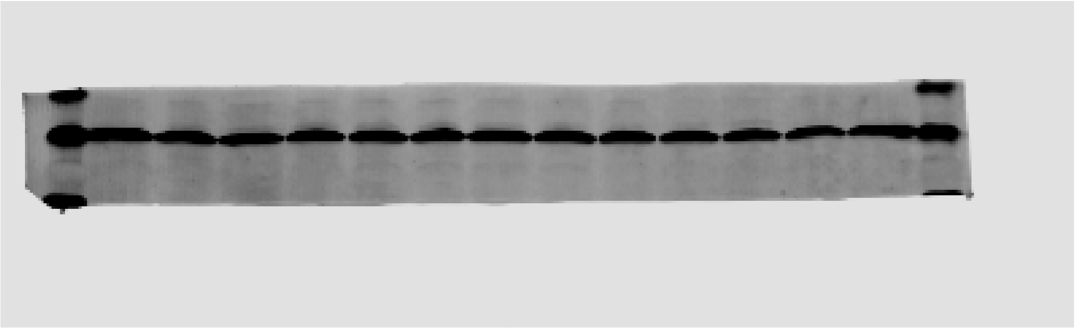

Fig4 C

Fig4 D

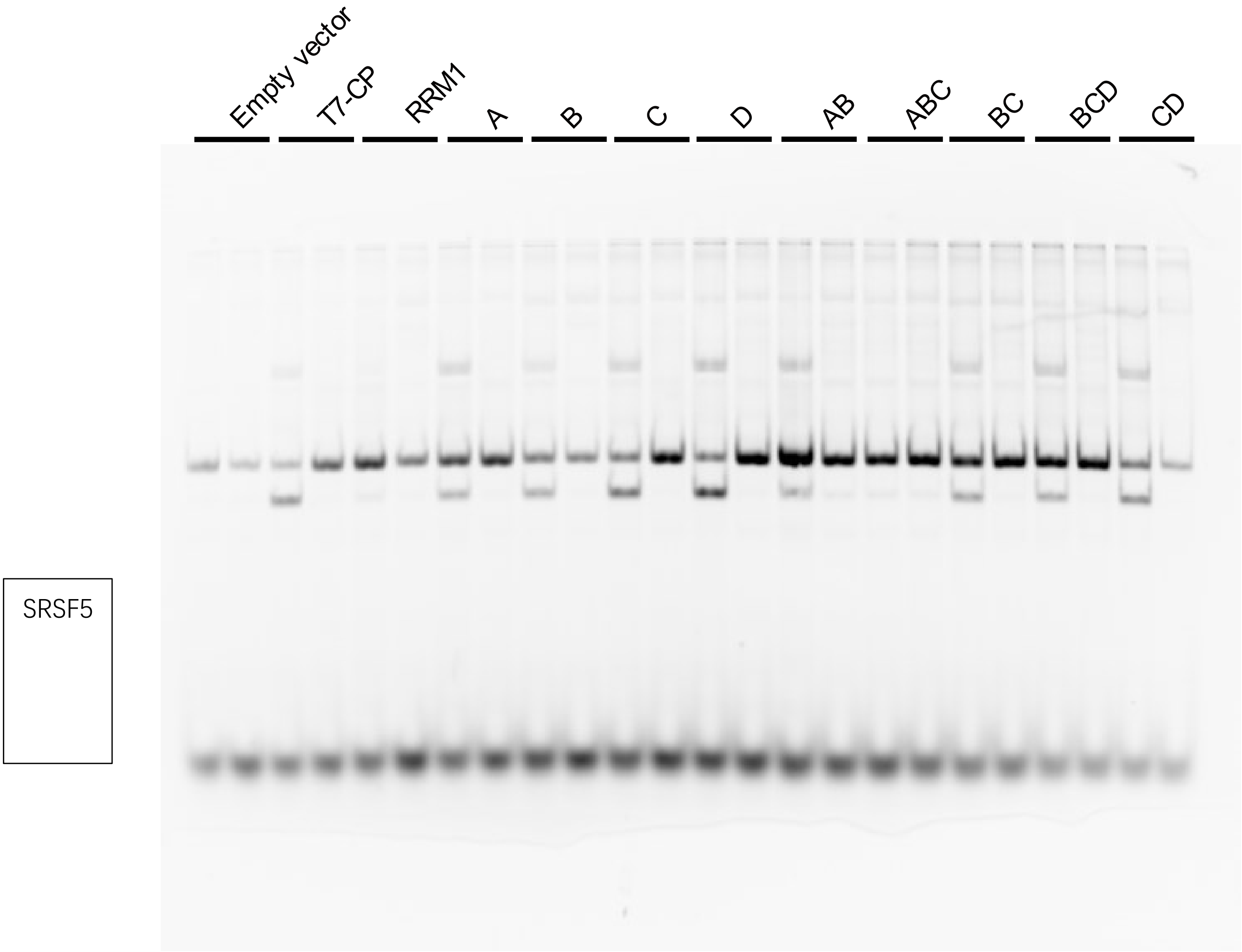
Empty vector
T7-CP
RRM1
A
B
C
D
AB
ABC
BC
BCD
CD
SRSF5

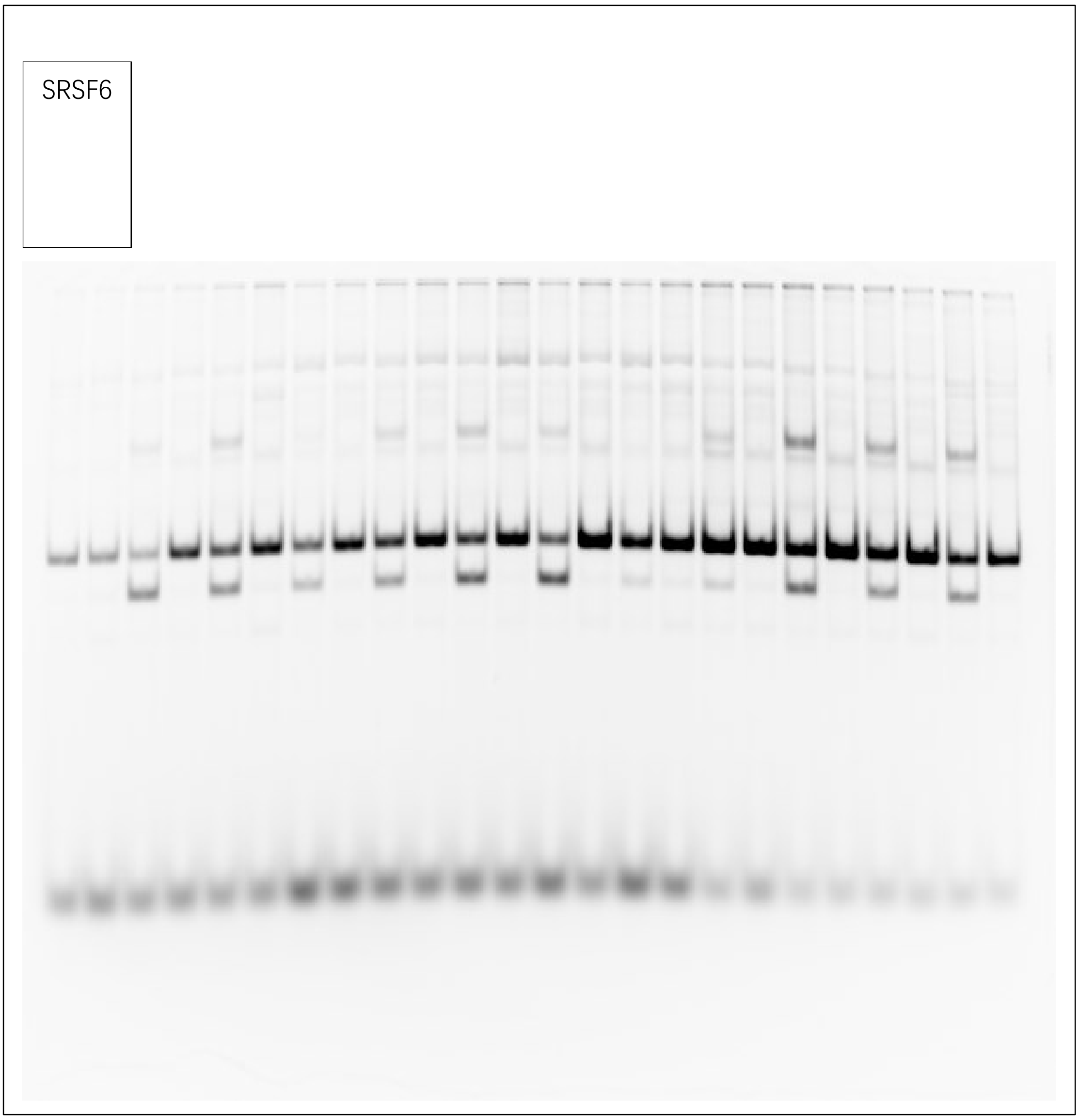

Fig5 A

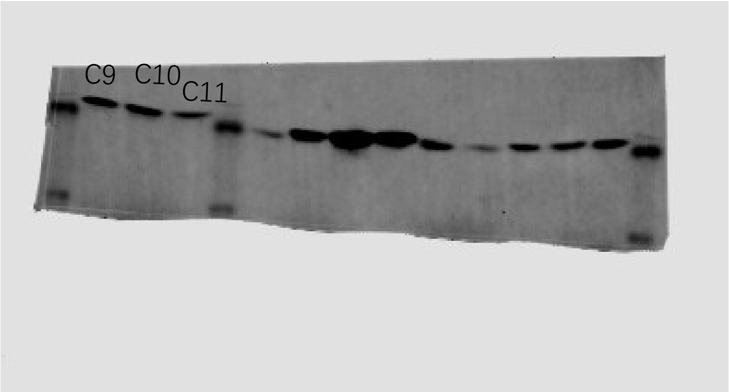

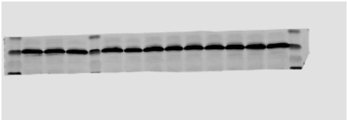

(Left)

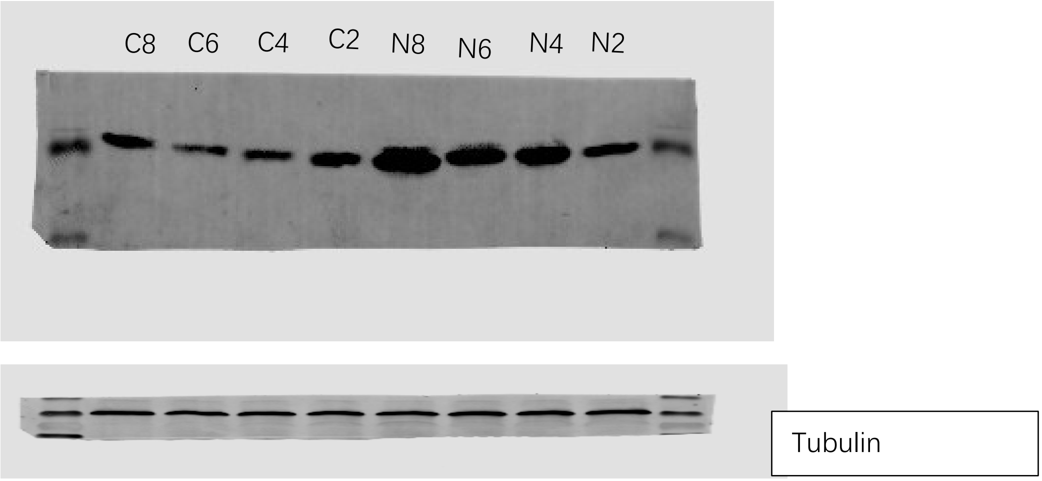
C8
C6
C4
C2
N8
N6
N4
N2
Tubulin

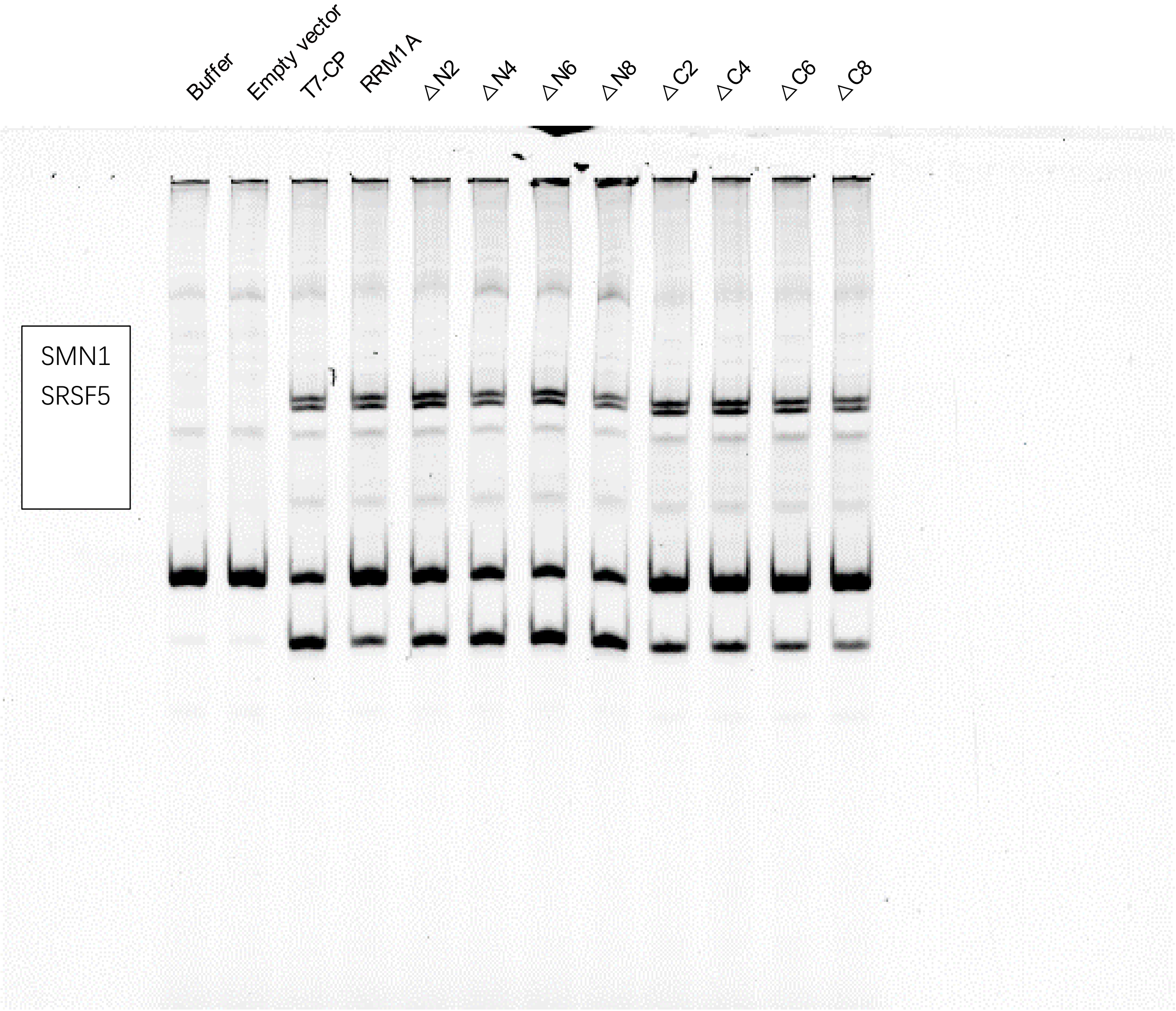
Buffer
Empty vector
T7-CP
RRM1 A
△N2
△N4
△N6
△N8
△C2
△C4
△C6
△C8
SMN1
SRSF5

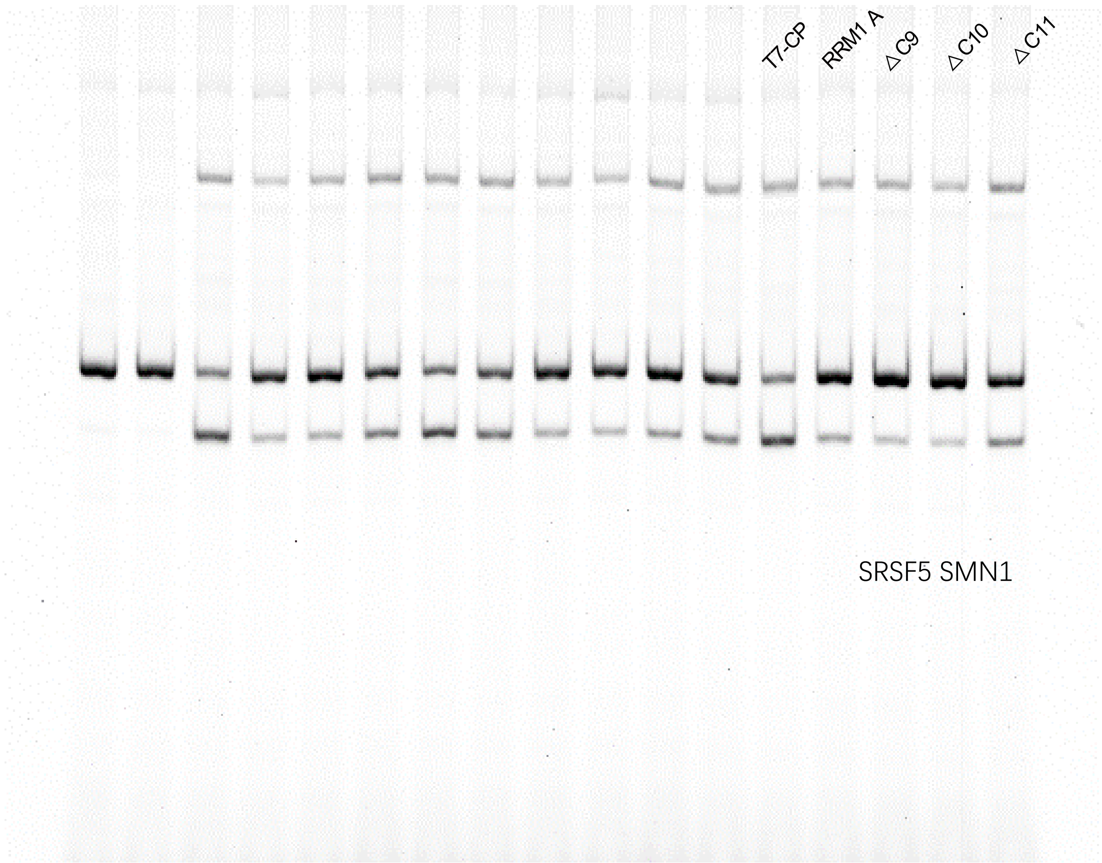

Fig5 B

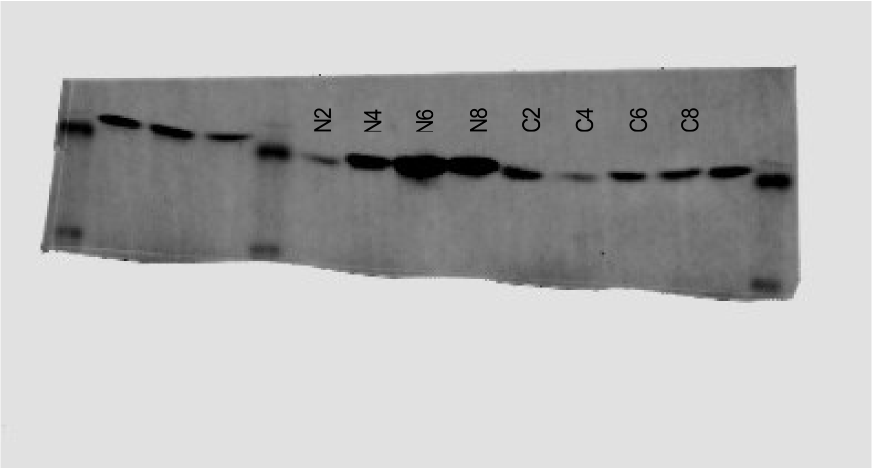

SRSF6

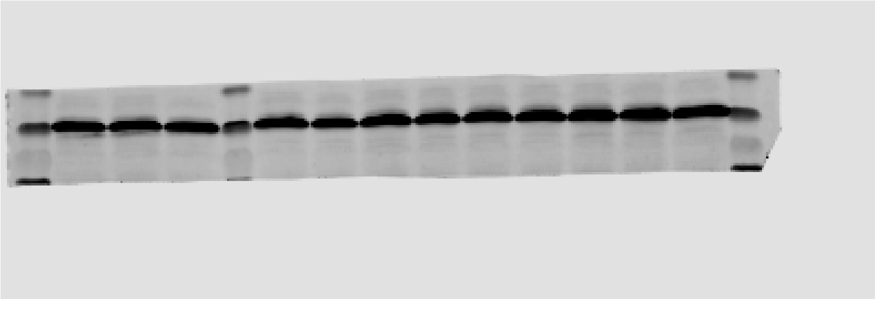

(Right)

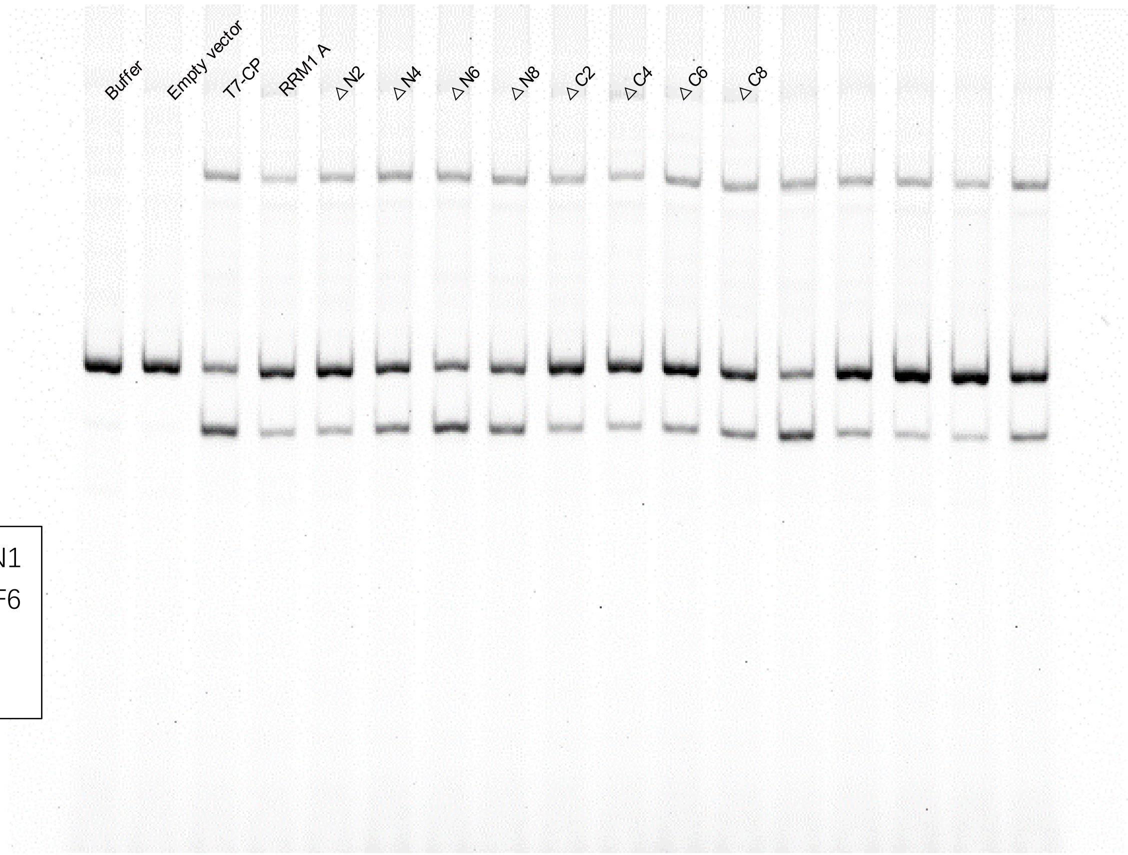

SMN1
SRSF6

(Left)
Fig5 C

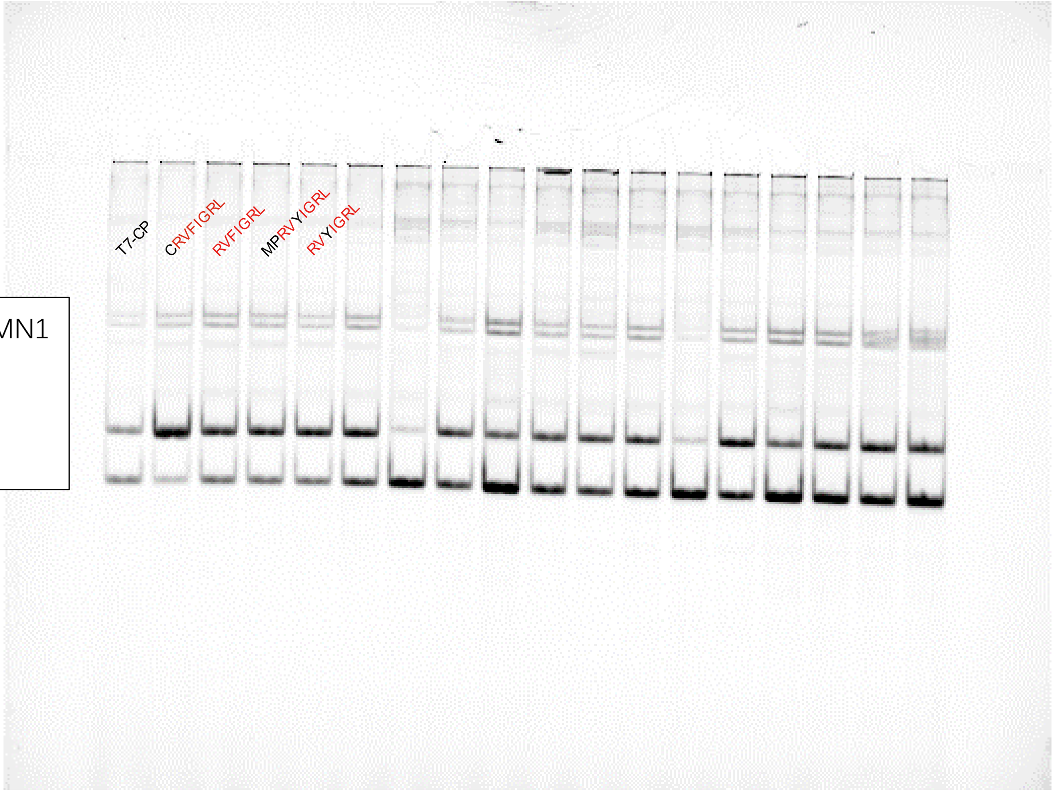

SMN1

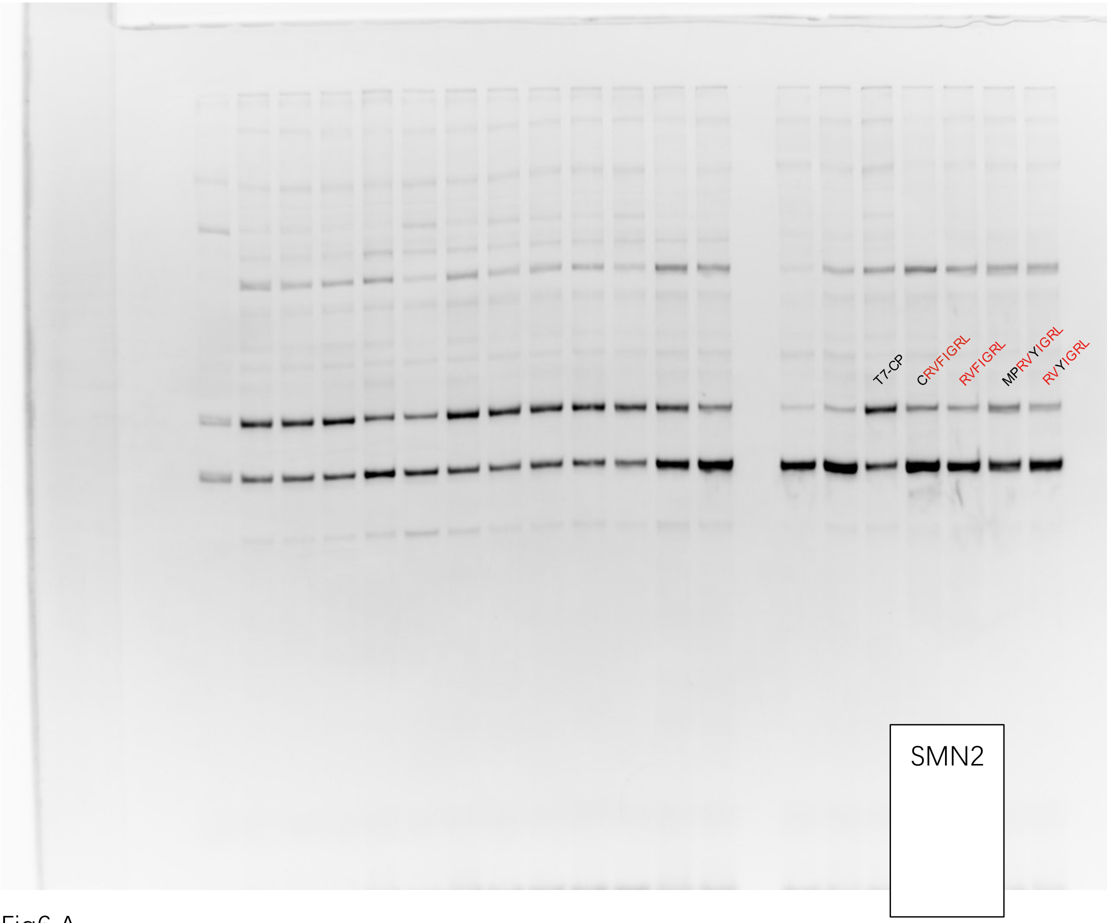

Fig6 A

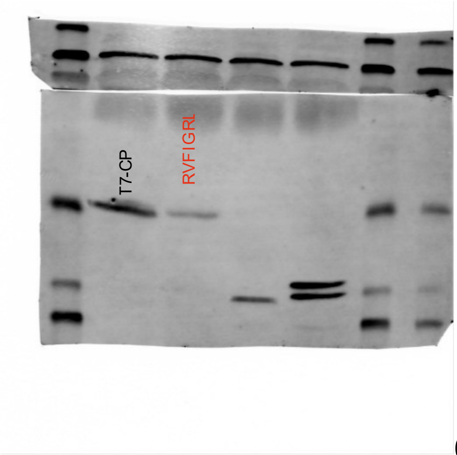

(Left)

Fig6 B

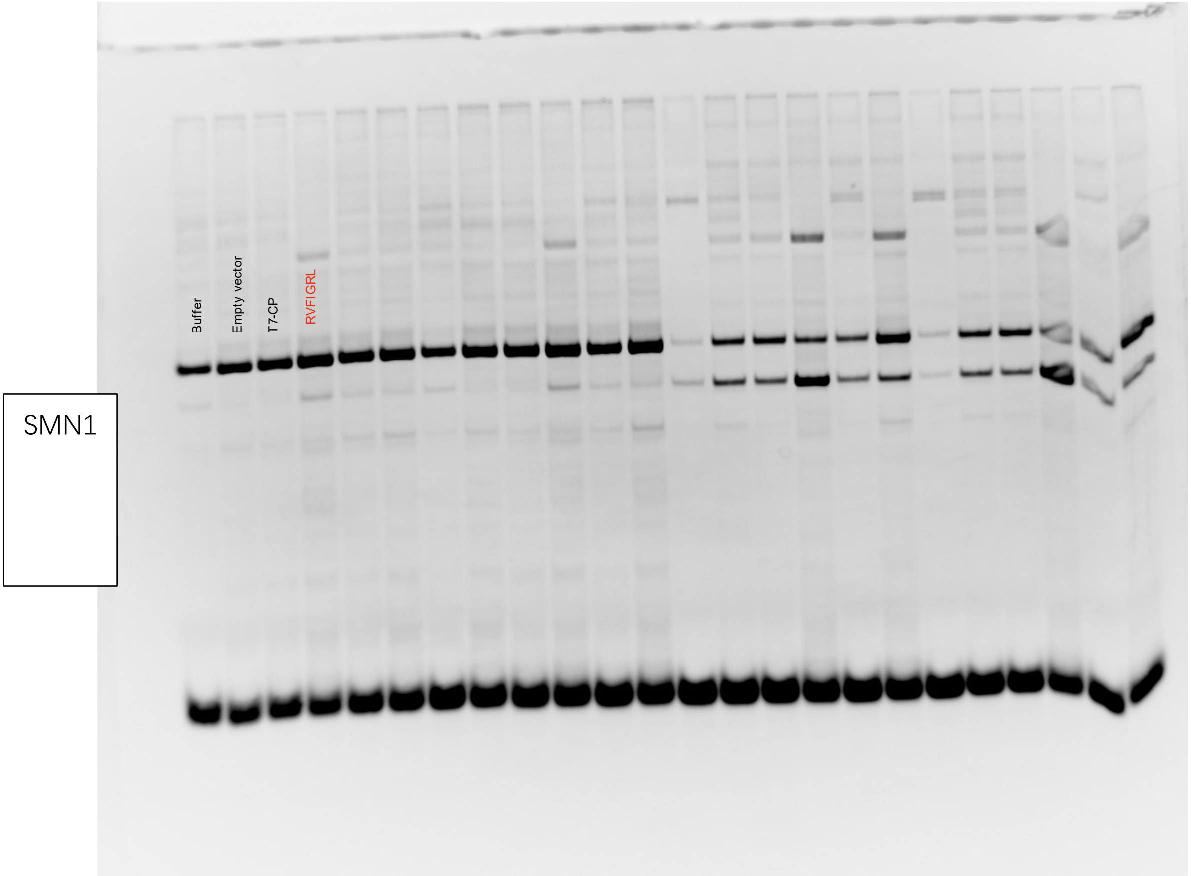
Buffer
Empty vector
T7-CP
RVFIGRL
SMN1

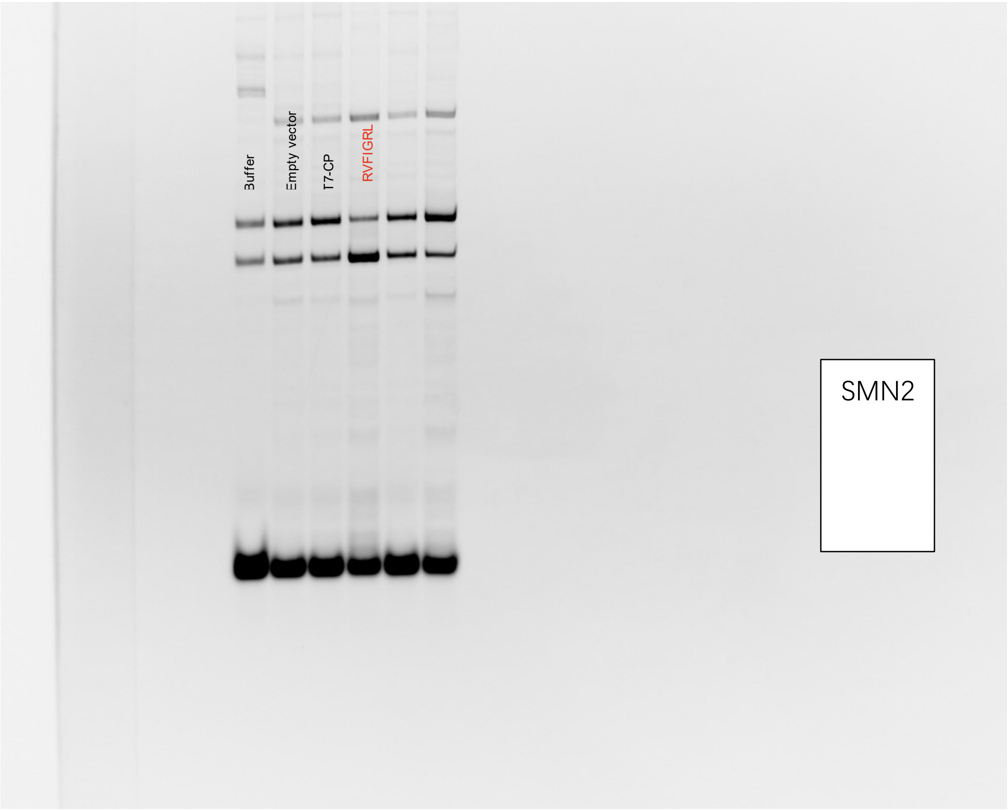
Buffer
Empty vector
T7-CP
RVFIGRL
SMN2

Fig6 C Fig6 D

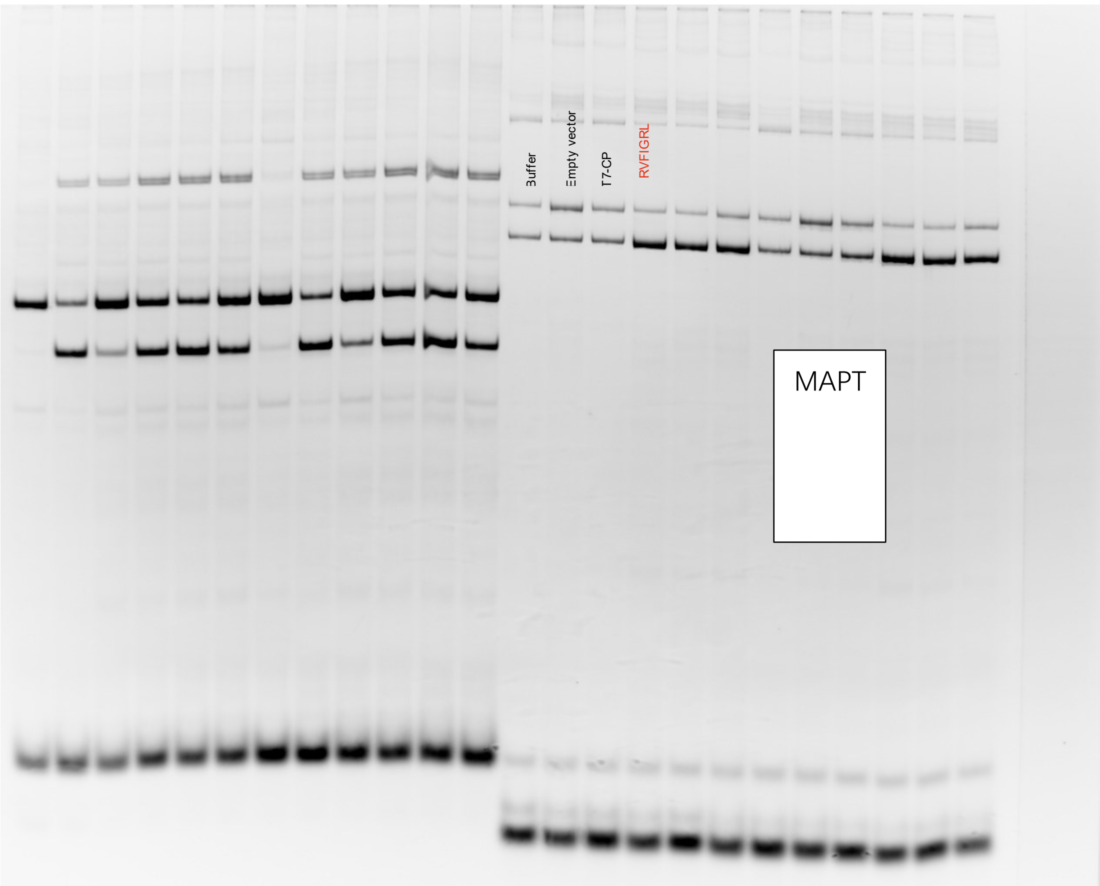
Buffer
Empty vector
T7-CP
RVFIGRL
MAPT

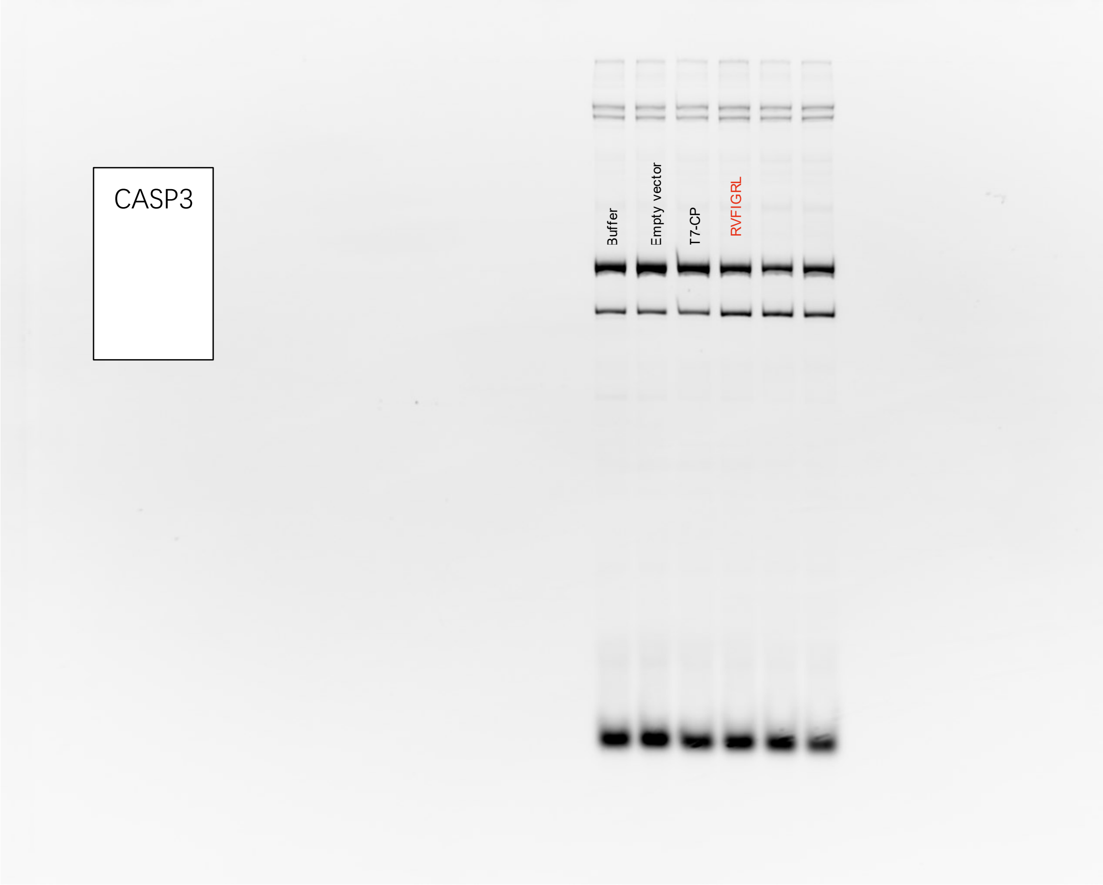
CASP3
Buffer
Empty vector
T7-CP
RVFIGRL

Figure S1

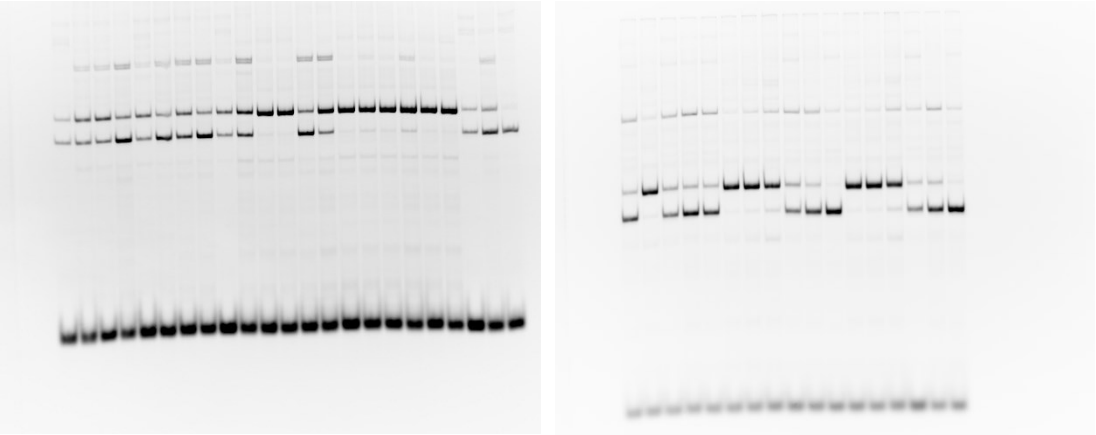

Figure S2 A

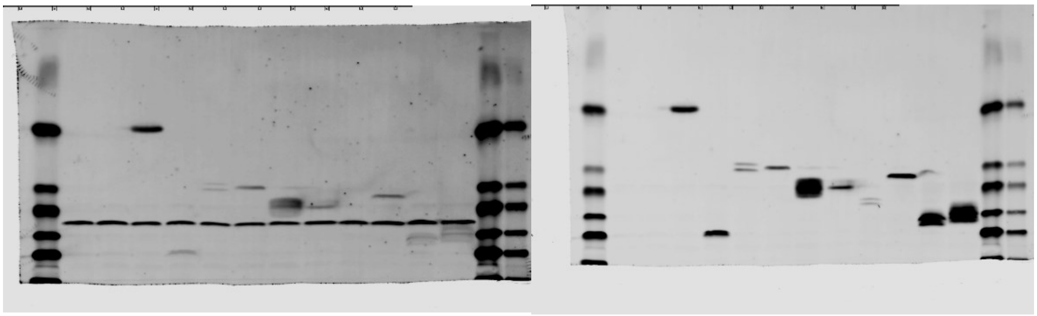

Figure S2 B

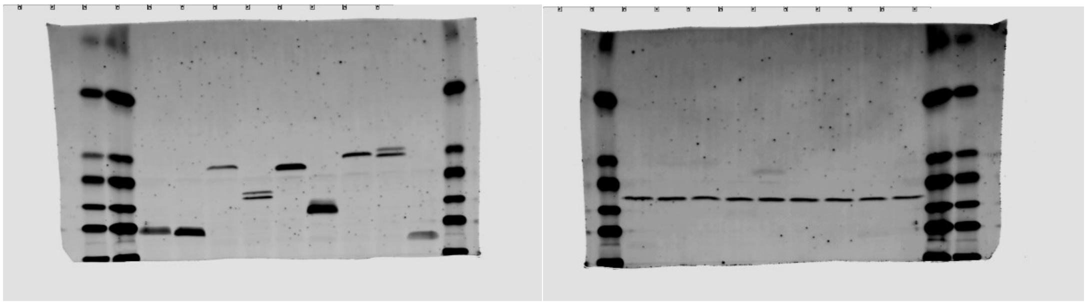

Figure S2 C

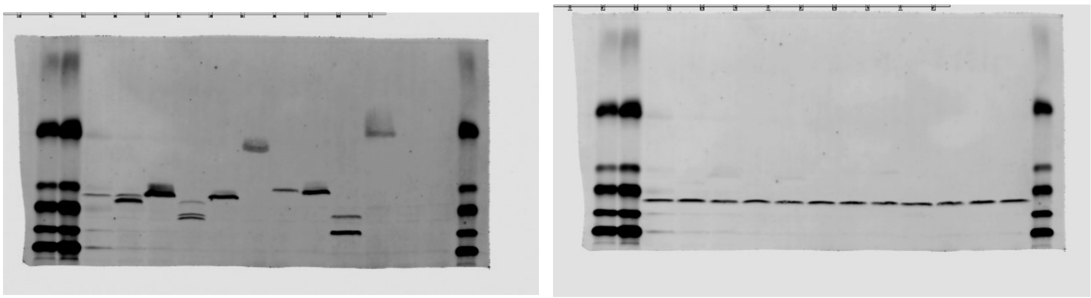

Figure S2 D

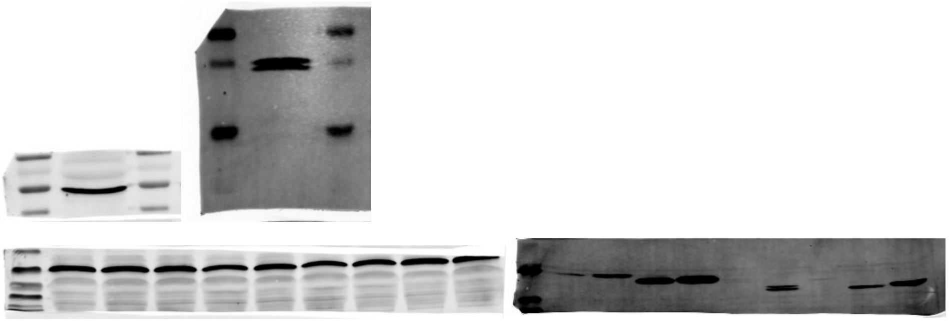

Figure S2 E

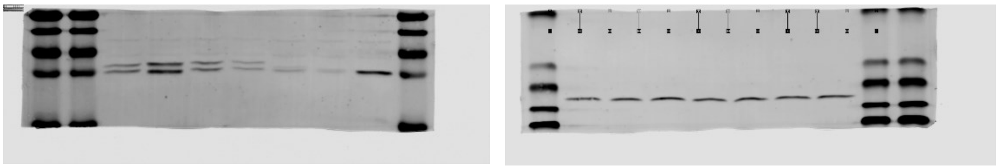

Figure S2 F

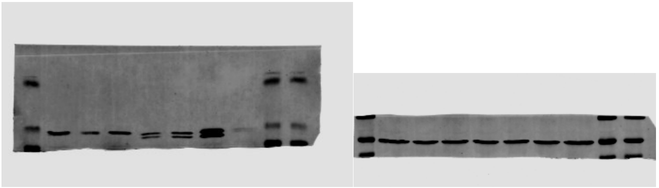

Figure S4

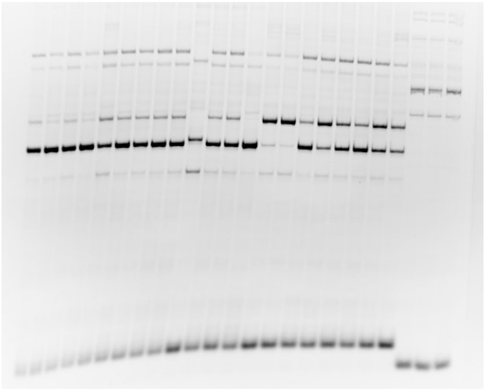

Figure S6

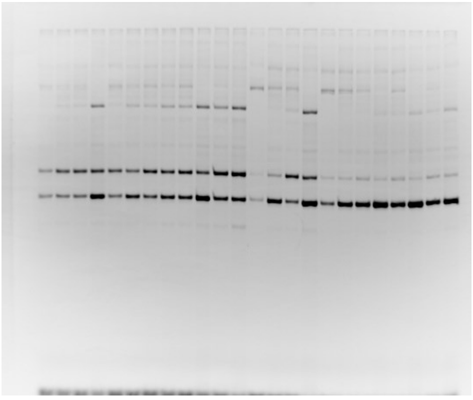

Supplement: Supplemental Information 1 [file peerj-11-16103-s001.pdf]
