# Supplementary material for: Identification of two short peptide motifs from serine/arginine-rich protein ribonucleic acid recognition motif-1 domain acting as splicing regulators
Source: PeerJ. 2023 Sep 18;11:e16103. doi: 10.7717/peerj.16103 (PMC10512959; doi:10.7717/peerj.16103)
Supplement: Supplemental Information 3 [file peerj-11-16103-s003.docx]

Supplementary Material

# Figure S1


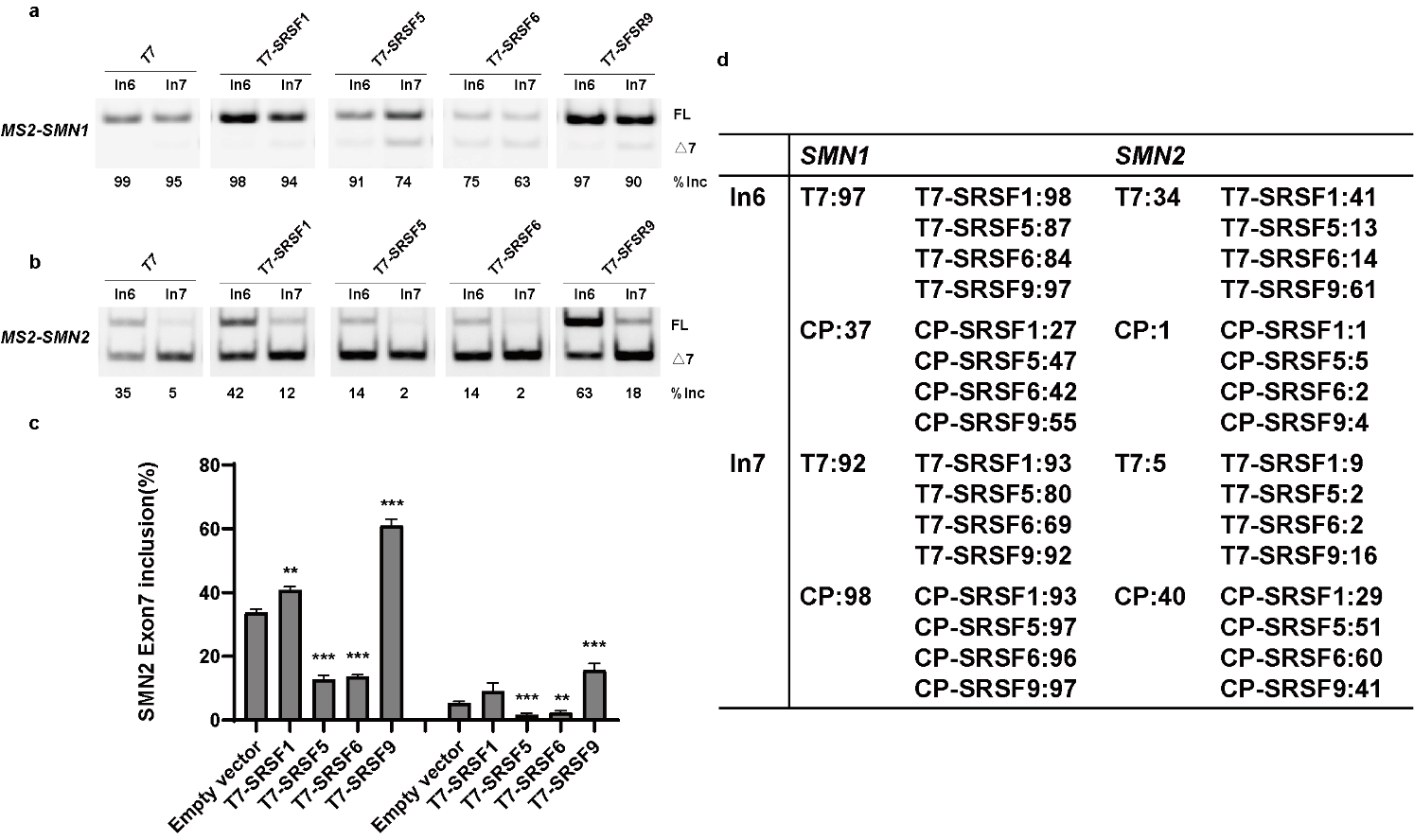


Figure S1 The splicing effects of four SR proteins (without CP)

(a-b) The functional analysis of free SR proteins on *MS2-SMN1/SMN2* minigene; (c) Quantitation of free proteins splicing data as shown in Figure 1c-d; (d) Visual statistics of all splicing data in Figure 1c-d.

# Figure S2


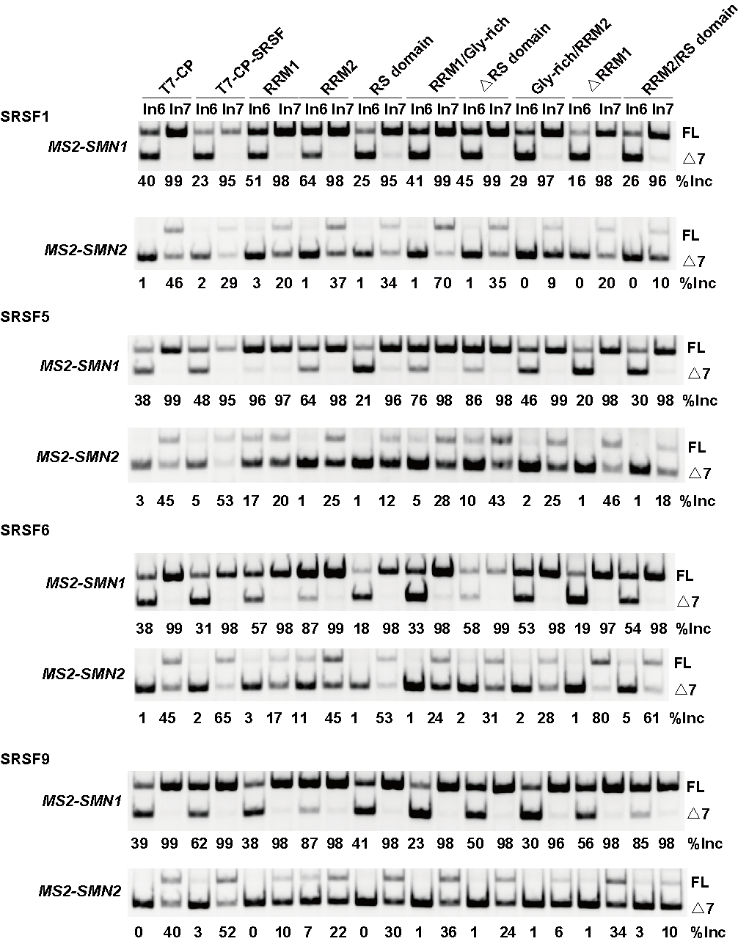


**Figure S2 The splicing effects of four SR protein domains**

# Figure S3


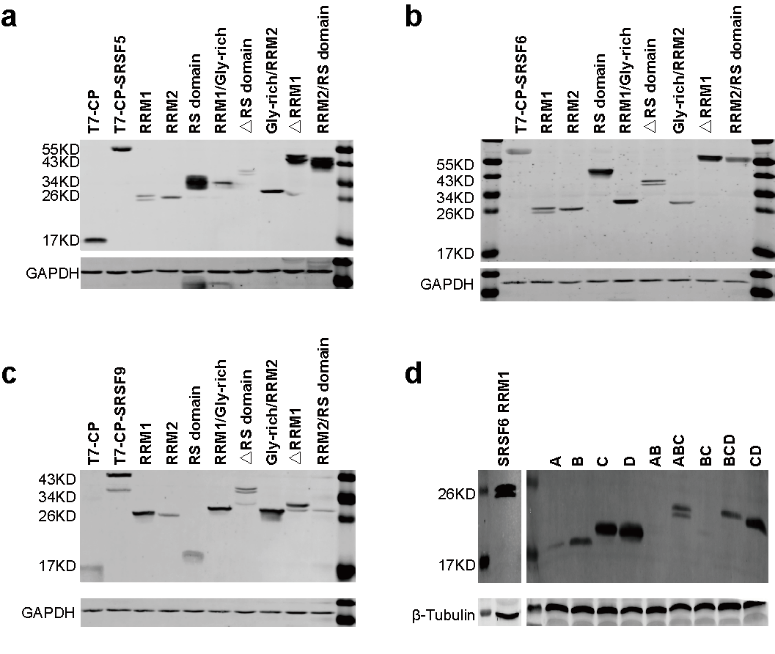


**Figure S3 Western blot of SR proteins and their truncated proteins**

(a) SRSF5 and its truncated proteins; (b) SRSF6 and its truncated proteins; (c) SRSF9 and its truncated proteins; (d) SRSF6 RRM1 and its related mutation proteins; (e) the charge-altered mutations of 9 N-terminal amino acids in SRSF1 RRM1; (f) the charge-altered mutations of 9 N-terminal amino acids in SRSF9 RRM1

# Figure S4


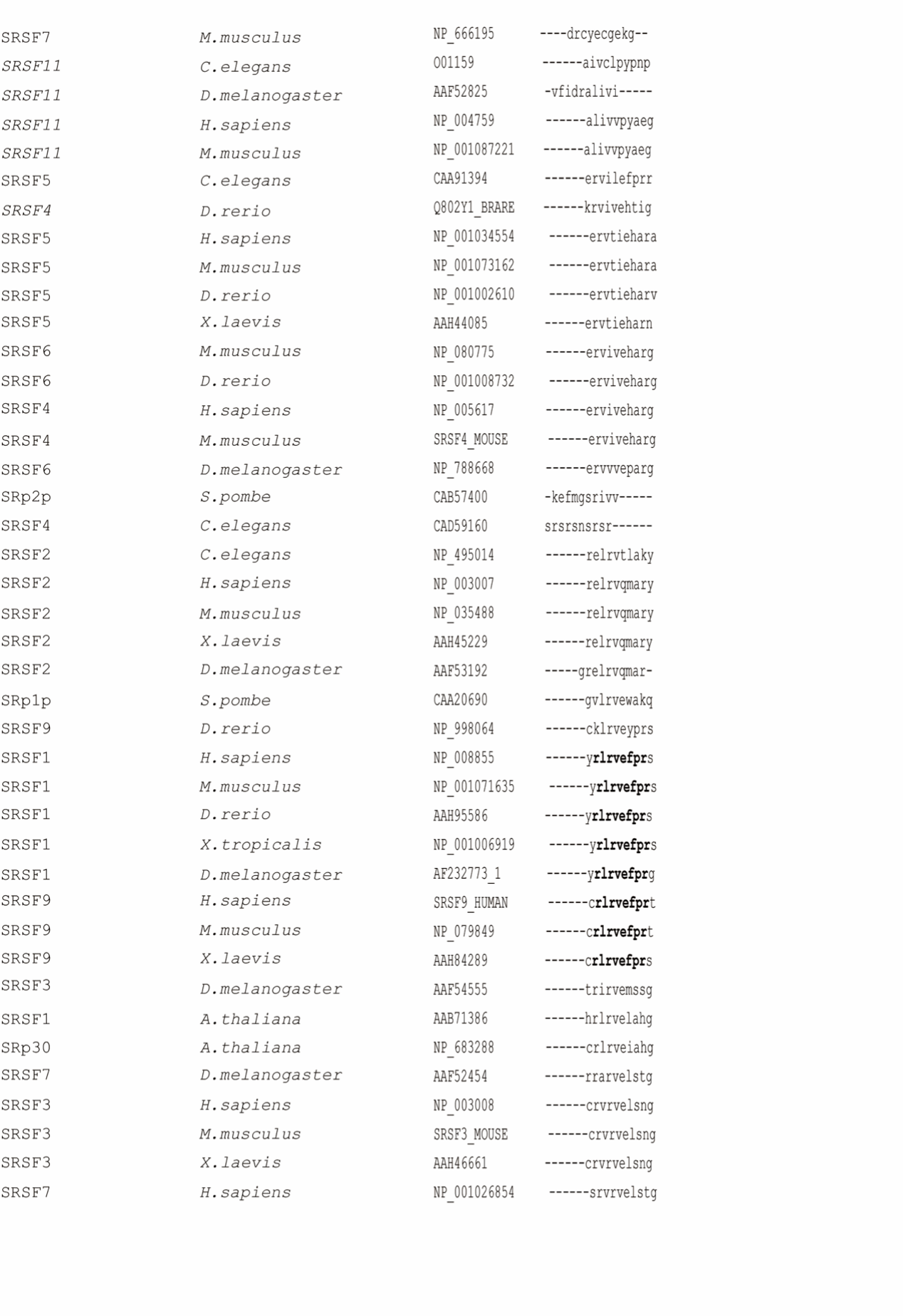


**Figure S4 Phylogenetic alignment of the C-termini of SRSF1/9 orthologs and paralogs**

# Figure S5


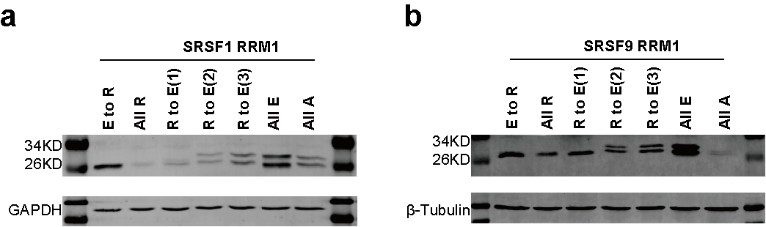


**Figure S5 Western blot of SR proteins and their truncated proteins**

(a) the charge-altered mutations of 9 N-terminal amino acids in SRSF1 RRM1; (b) the charge-altered mutations of 9 N-terminal amino acids in SRSF9 RRM1

# Figure S6


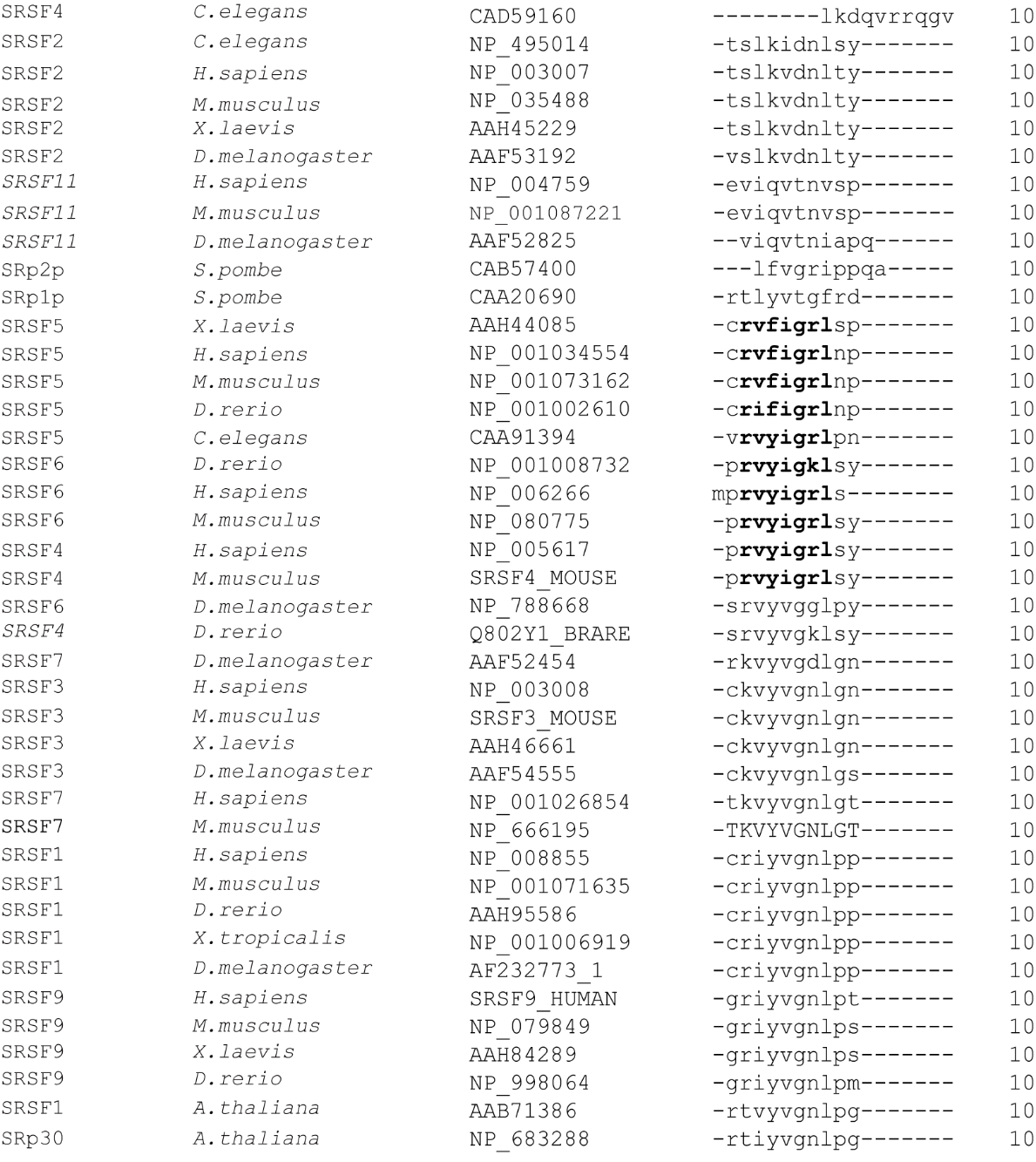


**Figure.S6 Phylogenetic alignment of the N-termini of SRSF5/6 RRM1 orthologs and paralogs**

# Figure S7


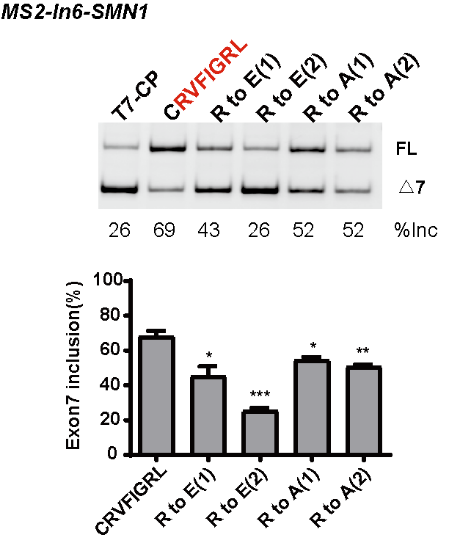


**Figure S7 The Arginine residue play key roles in the regulatory function of the short peptide motif**

Quantitative data from three independent experiments are shown in histograms. ***P < 0.001, **P < 0.01, *P < 0.05; A: Alanine, R: Arginine, E: Glutamicacid

# Figure S8


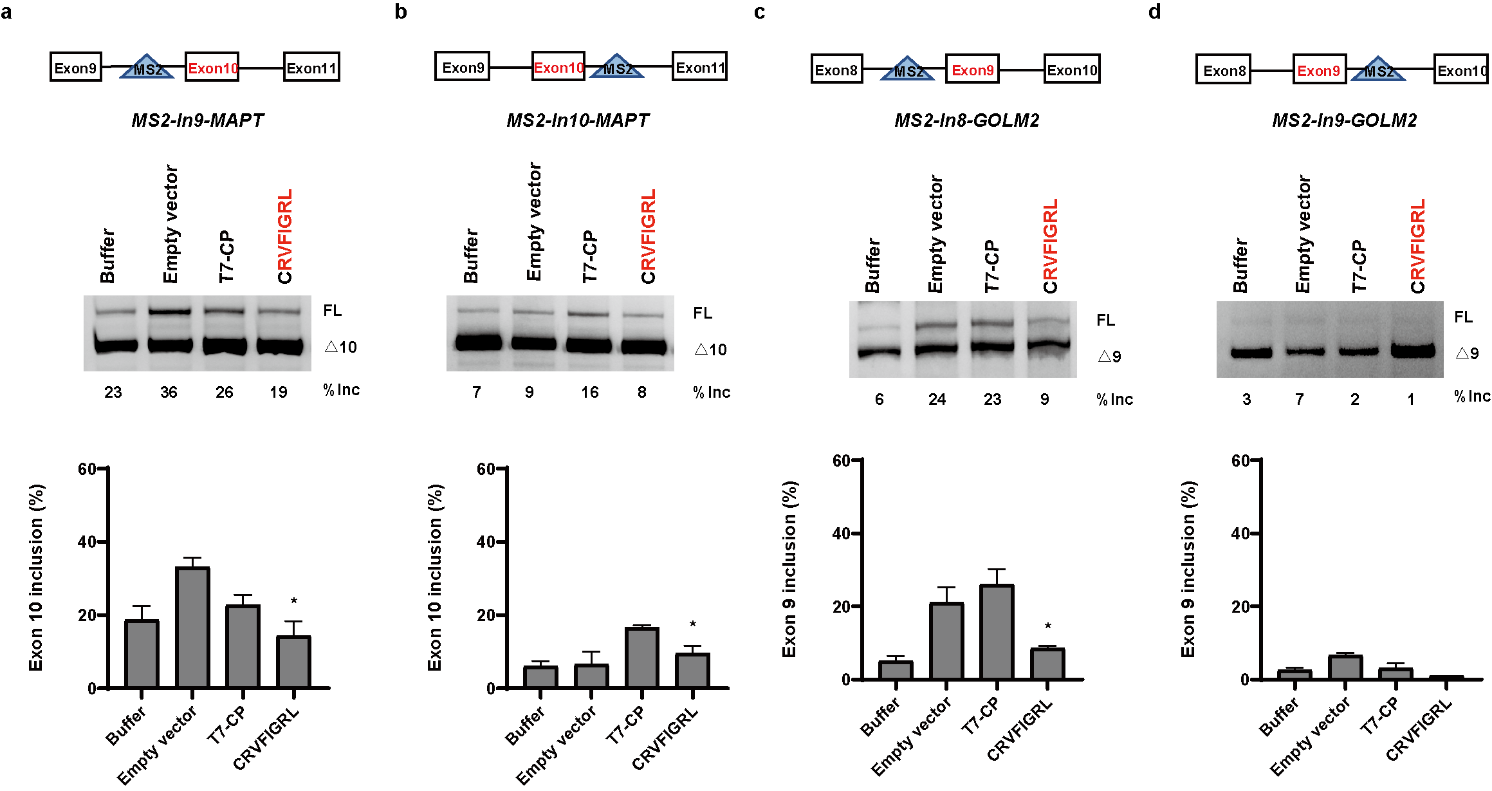


**Figure.S8 The short peptide motif displayed similar splicing effects on *MS2-MAPT*/*CASP3* minigenes**

Quantitative data from three independent experiments are shown in histograms. *P < 0.05;

# Figure S9


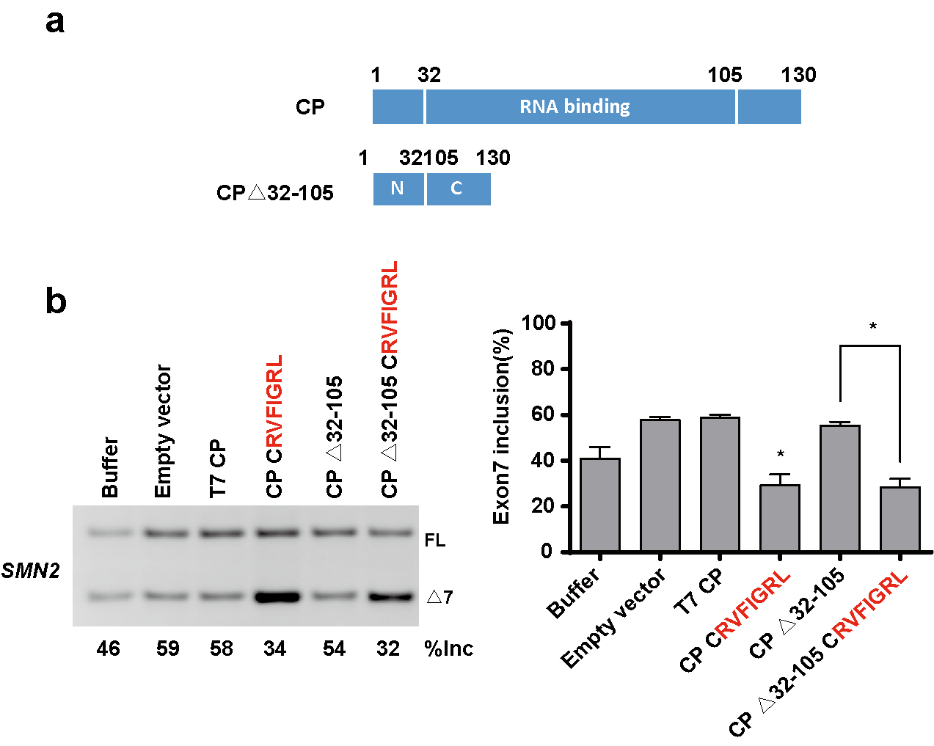


**Figure.S9 The short peptide play key roles in the regulatory splicing when deleted the RNA binding region of the CP**

(a) Diagram of the primary structure of the CP protein; (b) The splicing effects of the short peptide (CRVFIGRL) and its mutants on *SMN2* minigene. Quantitative data from three independent experiments are shown in histograms. *P < 0.05.

# Figure S10


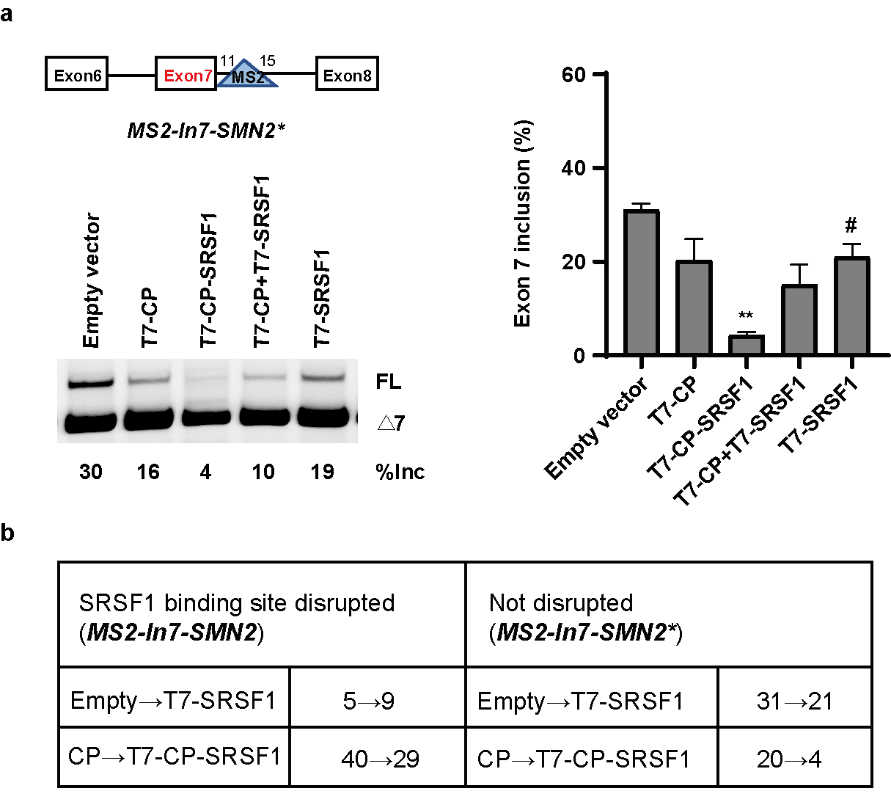


**Figure.S10 T7-SRSF1 and T7-CP-SRSF1 display inhibiting effects on *MS2-In7-SMN2** minigene**

Quantitative data from three independent experiments are shown in histograms. T7-CP+T7-SRSF1: 150 ng each for co-transfection with two expression plasmids. **P < 0.01 versus T7-CP; # P < 0.05 versus empty vector.
