# Supplementary material for: Identification of two short peptide motifs from serine/arginine-rich protein ribonucleic acid recognition motif-1 domain acting as splicing regulators
Source: PeerJ. 2023 Sep 18;11:e16103. doi: 10.7717/peerj.16103 (PMC10512959; doi:10.7717/peerj.16103)
Supplement: Supplemental Information 4 [file peerj-11-16103-s004.docx]

| **Names** | **Sequence (5′--> 3′)** |
| --- | --- |
| SLIC SRSF1 T7-F | CAGGTGGCCAACAGATGGGTatgtcgggaggtggtgtgattcg |
| SLIC SRSF1 T7-F | CCCAAACTCACCCTGAAGTTCTCATTATGTACGAGAGCGAGATCTG |
| SLIC SRSF5 T7-F | CAGGTGGCCAACAGATGGGTATGAGTGGCTGTCGGGTATTCATC |
| SLIC SRSF5 T7-R | CCCAAACTCACCCTGAAGTTCTCATTATGTACGAGAGCGAGATCTG |
| SLIC SRSF6 T7-F | CAGGTGGCCAACAGATGGGTATGCCGCGCGTCTACAT |
| SLIC SRSF6 T7-R | CCC AAA CTC ACC CTG AAG TTC TCA TTA ATC TCT GGA ACT CGA C |
| SLIC SRSF9 T7-F | CAGGTGGCCAACAGATGGGTATGTCGGGCTGGGCGGACGAG |
| SLIC SRSF9 T7-R | CCCAAACTCACCCTGAAGTTCTCATCAGTAGGGCCTGAAAGGAGAG |
| SLIC SRSF1 T7 MS2 -F | CAACTCCGGCATCTACgtcgacatgtcgggaggtggtgtgattcg |
| SLIC SRSF1 T7 MS2 -R | CCCAAACTCACCCTGAAGTTCTCATTATGTACGAGAGCGAGATCTG |
| SRSF5 T7 MS2 sal[I](http://nc2.neb.com/NEBcutter2/enz.php?name=d0323071-&enzname=KasI)-F | ACGTCGACATGAGTGGCTGTCGGG |
| SRSF5 T7 MS2 kpn[I](http://nc2.neb.com/NEBcutter2/enz.php?name=d0323071-&enzname=KasI)-R | ACGGTACCTTAATTGCCACTGTCAAC |
| SLIC SRSF6 T7 MS2 -F | CAACTCCGGCATCTACgtcgacATGCCGCGCGTCTACAT |
| SLIC SRSF6 T7 MS2 -R | CCC AAA CTC ACC CTG AAG TTC TCA TTA ATC TCT GGA ACT CGA C |
| SRSF9 T7 MS2 sal[I](http://nc2.neb.com/NEBcutter2/enz.php?name=d0323071-&enzname=KasI) -F | ACGTCGACATGTCGGGCTGGGC |
| SRSF9 T7 MS2 kpn[I](http://nc2.neb.com/NEBcutter2/enz.php?name=d0323071-&enzname=KasI) -R | ACGGTACCTCAGTAGGGCCTGAAAGGAG |

**Primers used for cloning SRSF into PCGT7 and PCGT7 MS2**

**Primers for generating T7 MS2-SRSF1 mutants**

| **Names** | **Sequence (5′--> 3′)** |
| --- | --- |
| SLIC 1 RRM1 -F | CAACTCCGGCATCTACgtcgacTGCCGCATCTACGTGGGTAACTTA |
| SLIC 1 RRM1 -R | CCCAAACTCACCCTGAAGTTCTCAGCTTCGAGGAAACTCCACCC |
| 1 RRM2 salΙ-F | ACG TCG ACAACA GAG TGG TTG TCT CTG GAC TGC |
| 1 RRM2 kpnΙ-R | ACG GTA CCC TAA TCA ACT TTA ACC CGG ATG TAG GC |
| 1 RS domain salΙ-F | ACG TCG ACAGAAGTCCAAGTTATGGAAGATCTCGATCT |
| 1 RS domain kpnΙ-R | ACG GTA CCT TAT GTA CGA GAG CGA GAT CTG CTA TGA |
| SLIC 1 RRM1/Gly-rich -F | CAACTCCGGCATCTACgtcgacTGCCGCATCTACGTGGGTAACTTA |
| SLIC 1 RRM1/Gly-rich -R | CCCAAACTCACCCTGAAGTTCTCAGCCATAGCGACCTCGGGGAG |
| SLIC 1 △RS domain -F | CAACTCCGGCATCTACgtcgacTGCCGCATCTACGTGGGTAACTTA |
| SLIC 1 △RS domain -R | CCCAAACTCACCCTGAAGTTCTCAATCAACTTTAACCCGGATGTAG |
| 1 Gly-rich/RRM2 salΙ-F | ACG TCG ACGGAACAGGCCGAGGCGGC |
| 1 Gly-rich/RRM2 kpnΙ-R | ACG GTA CCC TAA TCA ACT TTA ACC CGG ATG TAG GC |
| 1 △RRM1 salΙ-F | ACG TCG ACGGAACAGGCCGAGGCGGC |
| 1 △RRM1 kpnΙ-R | ACG GTA CCT TAT GTA CGA GAG CGA GAT CTG CTA TGA |
| 1 RRM2/RS domain salΙ-F | ACG TCG ACAACA GAG TGG TTG TCT CTG GAC TGC |
| 1 RRM2/RS domain kpnΙ-R | ACG GTA CCT TAT GTA CGA GAG CGA GAT CTG CTA TGA |
| SLIC 1 RRM1△C9-F | CAACTCCGGCATCTACgtcgacTGCCGCATCTACGTGGGTAACTTA |
| SLIC 1 RRM1△C9-R | CCCAAACTCACCCTGAAGTTCTCAGGTACCCATCGTAATCATAG |
| SLIC 1 RRM1+N-F | CAACTCCGGCATCTACgtcgacatgtcgggaggtggtgtgattcg |
| SLIC 1 RRM1+N-R | CCCAAACTCACCCTGAAGTTCTCAGCTTCGAGGAAACTCCACCC |
| SLIC 1 RRM1+N△C9-F | CAACTCCGGCATCTACgtcgacatgtcgggaggtggtgtgattcg |
| SLIC 1 RRM1+N△C9-R | CCCAAACTCACCCTGAAGTTCTCAGGTACCCATCGTAATCATAG |
| 1 RRM1 E→R-F | TACCGTCTGCGGGTGCGTTTTCCTCGAAG |
| 1 RRM1 E→R-R | CAC CCG CAG ACG GTA CCC ATC GTA ATC ATA |
| 1 RRM1 ALL R-F | TACGATGGGTACCGT CGTCGTCGTCGTCGTCGTCGTCGTtgagaacttc |
| 1 RRM1 ALL R-R | ACG GTA CCC ATC GTA ATC ATA GCC GTC GCG AC |
| 1 RRM1 R→E①-F | GATTACGATGGGTACGAGCTGCGGGTGGAG |
| 1 RRM1 R→E①-R | GTA CCC ATC GTA ATC ATA GCC GTC GCG ACC A |
| 1 RRM1 R→E②-F | GATGGGTACGAGCTG GAGGTG GAG TTT CCT |
| 1 RRM1 R→E②-R | CAG CTC GTA CCC ATC GTA ATC ATA GCC GTC G |
| 1 RRM1 R→E③-F | GAG GTG GAG TTT CCTGAGAGCtgagaac |
| 1 RRM1 R→E③-R | AGG AAA CTC CAC CTC CAG CTC GTA CCC ATC |
| 1 RRM1 ALL E-F | GATTACGATGGGTACGAGGAGGAGGAGGAGGAGGAGGAGGAGtgagaacttc |
| 1 RRM1 ALL E-R | GTA CCC ATC GTA ATC ATA GCC GTC GCG ACC ATA CA |
| 1 RRM1 ALL A-F | GATTACGATGGGTAC GCTGCTGCTGCTGCTGCTGCTGCTGCT tgagaacttc |
| 1 RRM1 ALL A-R | GTA CCC ATC GTA ATC ATA GCC GTC GCG ACC ATA CA |

**Primers for generating T7 MS2-SRSF5 mutants**

| **Names** | **Sequence (5′--> 3′)** |
| --- | --- |
| 5 RRM1 SalΙ-F | ACGTCGACTGTCGGGTATTCATC |
| 5 RRM1 KpnΙ-F | ACGGTACCC TAAGC CCT AGC ATG TTC AAT AGT AAC |
| 5 RRM2 salΙ-F | ACGTCGACAATCGTCTTATAGTTGAGAATTTATCCTC |
| 5 RRM2 kpnΙ-R | ACGGTACCC TA TTT GCT GCC TTC AAT TAA TTT |
| 5 RS domain salΙ-F | ACGTCGACAGGCACAGTAGGTCAAGAAGCAGGTCT |
| 5 RS domain kpnΙ-R | ACGGTACC TTA ATT GCC ACT GTC AAC TGA TCT GGA C |
| 5 RRM1/Gly-rich salΙ-F | ACGTCGACTGTCGGGTATTCATC |
| 5 RRM1/Gly-rich kpnΙ-R | ACGGTACCC TA TTC TGT TCT TAC AGG TGG AGC A |
| 5 △RS domain salΙ-F | ACGTCGACTGTCGGGTATTCATC |
| 5 △RS domain kpnΙ-R | ACGGTACCC TA TTT GCT GCC TTC AAT TAA TTT |
| 5 Gly-rich/RRM2 salΙ-F | ACGTCGACGGTGGAAGAGGTAGAGGACGATACTCTGAC |
| 5 Gly-rich/RRM2 kpnΙ-R | ACGGTACCC TA TTT GCT GCC TTC AAT TAA TTT |
| 5 △RRM1 salΙ-F | ACGTCGACGGTGGAAGAGGTAGAGGACGATACTCTGAC |
| 5 △RRM1 kpnΙ-R | ACGGTACC TTA ATT GCC ACT GTC AAC TGA TCT GGA C |
| 5 RRM2/RS domain salΙ-F | ACGTCGACAATCGTCTTATAGTTGAGAATTTATCCTC |
| 5 RRM2/RS domain kpnΙ-R | ACGGTACC TTA ATT GCC ACT GTC AAC TGA TCT GGA C |
| SLIC 5A-F | CAACTCCGGCATCTACgtcgacTGTCGGGTATTCATCGGGAG |
| SLIC 5A-R | CCC AAA CTC ACC CTG AAG TTC TCA TTC CAC GTC CTT CTC CCT G |
| SLIC 5B-F | CAACTCCGGCATCTACgtcgacAGATTCTTCAAGGGATATG |
| SLIC 5B-R | CCC AAA CTC ACC CTG AAG TTC TCA AAA GCC TCT TTT CAG ATC A |
| SLIC 5C-F | CAACTCCGGCATCTACgtcgacGGTTTTGTGGAATTTGAGGA |
| SLIC 5C-R | CCC AAA CTC ACC CTG AAG TTC TCA AAG CTC ATA CAC AGC ATC A |
| SLIC 5D-F | CAACTCCGGCATCTACgtcgacGATGGAAAAGAACTCTGTAG |
| SLIC 5D-R | CCC AAA CTC ACC CTG AAG TTC TCA AGC CCT AGC ATG TTC AAT A |
| SLIC 5AB-F | CAACTCCGGCATCTACgtcgacTGTCGGGTATTCATCGGGAG |
| SLIC 5AB-R | CCC AAA CTC ACC CTG AAG TTC TCA AAA GCC TCT TTT CAG ATC A |
| SLIC 5ABC-F | CAACTCCGGCATCTACgtcgacTGTCGGGTATTCATCGGGAG |
| SLIC 5ABC-R | CCC AAA CTC ACC CTG AAG TTC TCA AAG CTC ATA CAC AGC ATC A |
| SLIC 5BC-F | CAACTCCGGCATCTACgtcgacAGATTCTTCAAGGGATATG |
| SLIC 5BC-R | CCC AAA CTC ACC CTG AAG TTC TCA AAG CTC ATA CAC AGC ATC A |
| SLIC 5BCD-F | CAACTCCGGCATCTACgtcgacAGATTCTTCAAGGGATATG |
| SLIC 5BCD -R | CCC AAA CTC ACC CTG AAG TTC TCA AGC CCT AGC ATG TTC AAT A |
| SLIC 5CD -F | CAACTCCGGCATCTACgtcgacGGTTTTGTGGAATTTGAGGA |
| SLIC 5CD-R | CCC AAA CTC ACC CTG AAG TTC TCA AGC CCT AGC ATG TTC AAT A |
| 5A△C2-F | GCC AGGGAG AAGGAC tgagaacttca |
| 5A△C2-R | GTC CTT CTC CCT GGC CGC TGG ATT TAG TCT |
| 5A△C4-F | CCA GCGGCC AGGGAG tgagaacttca |
| 5A△C4-R | CTC CCT GGC CGC TGG ATT TAG TCT CCC GAT |
| 5A△C6-F | CTA AATCCA GCGGCC tgagaacttca |
| 5A△C6-R | GGC CGC TGG ATT TAG TCT CCC GAT GAA TAC |
| 5A△C8-F | GGG AGACTA AATCCA tgagaacttca |
| 5A△C8-R | TGG ATT TAG TCT CCC GAT GAA TAC CCG ACA |
| 5A△C9-F | ATCGGG AGACTA AAT tgagaacttca |
| 5A△C9-R | ATT TAG TCT CCC GAT GAA TAC CCG ACA GTC |
| 5A△C10-F | TTC ATCGGG AGACTA tgagaacttca |
| 5A△C10-R | TAG TCT CCC GAT GAA TAC CCG ACA GTC GAC |
| 5A△C11-F | GTATTC ATCGGG AGA tgagaacttca |
| 5A△C11-R | TCT CCC GAT GAA TAC CCG ACA GTC GAC GTA |
| 5A△C10△N1-F | GGCATCTACGTCGAC CGG GTATTC A |
| 5A△C10△N1-R | GTC GAC GTA GAT GCC GGA GTT GGC GGC GAT G |
| 5A△N2-F | GGCATCTACGTCGAC GTATTC ATCGG |
| 5A△N2-R | GTC GAC GTA GAT GCC GGA GTT GGC GGC GAT G |
| 5A△N4-F | GGCATCTACGTCGAC ATCGGG AGACT |
| 5A△N4-R | GTC GAC GTA GAT GCC GGA GTT GGC GGC GAT G |
| 5A△N6-F | GGCATCTACGTCGAC AGACTA AATCCA GCG |
| 5A△N6-R | GTC GAC GTA GAT GCC GGA GTT GGC GGC GAT G |
| 5A△N8-F | GGCATCTACGTCGAC AATCCA GCGG |
| 5A△N8-R | GTC GAC GTA GAT GCC GGA GTT GGC GGC GAT G |

**Primers for generating T7 MS2-SRSF6 mutants**

| **Names** | **Sequence (5′--> 3′)** |
| --- | --- |
| SLIC 6 RRM1 -F | CAACTCCGGCATCTACgtcgacATGCCGCGCGTCTACATAGGAC |
| SLIC 6 RRM1 -R | CCC AAA CTC ACC CTG AAG TTC TCA GCC CCG GGC GTG CTC TAC GAT CA |
| SLIC 6 RRM2 -F | CAACTCCGGCATCTACgtcgacTACAGGCTTATTGTAGAAAATC |
| SLIC 6 RRM2 -R | CCC AAA CTC ACC CTG AAG TTC TCA TGG CTT ATC TTC AAT AAG CC |
| 6 RS domain salΙ-F | ACGTCGACCGCACAAGCCATAGGCGATCTTACTCTGGA |
| 6 RS domain BamhΙ-R | ACG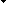GATC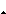C TTA ATC TCT GGA ACT CGA CCT GGA CCT TG |
| SLIC 6 RRM1/Gly-rich -F | CAACTCCGGCATCTACgtcgacATGCCGCGCGTCTACATAGGAC |
| SLIC 6 RRM1/Gly-rich -R | CCC AAA CTC ACC CTG AAG TTC TCA TCC ACC TCC ACC ACT GCG GCT T |
| SLIC 6 △RS domain -F | CAACTCCGGCATCTACgtcgacATGCCGCGCGTCTACATAGGAC |
| SLIC 6 △RS domain -R | CCC AAA CTC ACC CTG AAG TTC TCA TGG CTT ATC TTC AAT AAG CC |
| SLIC 6 Gly-rich/RRM2 -F | CAACTCCGGCATCTACgtcgacGGTGGAGGTGGATACAGCAG |
| SLIC 6 Gly-rich/RRM2 -R | CCC AAA CTC ACC CTG AAG TTC TCA TGG CTT ATC TTC AAT AAG CC |
| SLIC 6 △RRM1 -F | CAACTCCGGCATCTACgtcgacGGTGGAGGTGGATACAGCAG |
| SLIC 6 △RRM1 -R | CCC AAA CTC ACC CTG AAG TTC TCA TTA ATC TCT GGA ACT CGA C |
| SLIC 6 RRM2/RS domain -F | CAACTCCGGCATCTACgtcgacTACAGGCTTATTGTAGAAAATC |
| SLIC 6 RRM2/RS domain -R | CCC AAA CTC ACC CTG AAG TTC TCA TTA ATC TCT GGA ACT CGA C |
| SLIC 6A-F | CAACTCCGGCATCTACgtcgacATGCCGCGCGTCTACAT |
| SLIC 6A-R | CCC AAA CTC ACC CTG AAG TTC TCA GAT GTC CTT CTC CCG GAC GT |
| SLIC 6B-F | CAACTCCGGCATCTACgtcgacCAGCGCTTTTTCAGTGGCTA |
| SLIC 6B-R | CCCAAACTCACCCTGAAGTTCTCACCCATTTTTGAGGTCTAC |
| SLIC 6C-F | CAACTCCGGCATCTACgtcgacTACGGCTTCGTGGAGTTCG |
| SLIC 6C-R | CCC AAA CTC ACC CTG AAG TTC TCA CTC GTA AAC GGC GTC GTC |
| SLIC 6D-F | CAACTCCGGCATCTACgtcgacCTGAACGGCAAGGAGCTC |
| SLIC 6D-R | CCC AAA CTC ACC CTG AAG TTC TCA GCC CCG GGC GTG CTC TA |
| SLIC 6AB-F | CAACTCCGGCATCTACgtcgacATGCCGCGCGTCTACAT |
| SLIC 6AB-R | CCC AAA CTC ACC CTG AAG TTC TCA CCC ATT TTT GAG GTC TAC T |
| SLIC 6ABC-F | CAACTCCGGCATCTACgtcgacATGCCGCGCGTCTACAT |
| SLIC 6ABC-R | CCC AAA CTC ACC CTG AAG TTC TCA CTC GTA AAC GGC GTC GTC |
| SLIC 6BC-F | CAACTCCGGCATCTACgtcgacCAGCGCTTTTTCAGTGGCTA |
| SLIC 6BC-R | CCC AAA CTC ACC CTG AAG TTC TCA CTC GTA AAC GGC GTC GTC |
| SLIC 6BCD-F | CAACTCCGGCATCTACgtcgacCAGCGCTTTTTCAGTGGCTA |
| SLIC 6BCD -R | CCC AAA CTC ACC CTG AAG TTC TCA GCC CCG GGC GTG CTC TA |
| SLIC 6CD -F | CAACTCCGGCATCTACgtcgacTACGGCTTCGTGGAGTTCG |
| SLIC 6CD-R | CCC AAA CTC ACC CTG AAG TTC TCA GCC CCG GGC GTG CTC TA |
| 6A△N2-F | GGCATCTACGTCGAC CGCGTC TACATA |
| 6A△N2-R | GTC GAC GTA GAT GCC GGA GTT GGC GGC GAT G |
| 6A△N4-F | GGCATCTACGTCGAC TACATA GGACGC |
| 6A△N4-R | GTC GAC GTA GAT GCC GGA GTT GGC GGC GAT G |
| 6A△N6-F | GGCATCTACGTCGAC GGACGC CTGAGC |
| 6A△N6-R | GTC GAC GTA GAT GCC GGA GTT GGC GGC GAT G |
| 6A△N8-F | GGCATCTACGTCGAC CTGAGC TACAAC |
| 6A△N8-R | GTC GAC GTA GAT GCC GGA GTT GGC GGC GAT G |
| 6A△C2-F | AAC GTCCGG GAGAAG tgagaacttca |
| 6A△C2-R | CTT CTC CCG GAC GTT GTA GCT CAG GCG TCC |
| 6A△C4-F | AGC TACAAC GTCCGG tgagaacttca |
| 6A△C4-R | CCG GAC GTT GTA GCT CAG GCG TCC TAT GTA |
| 6A△C6-F | CGC CTGAGC TACAAC tgagaacttca |
| 6A△C6-R | GTT GTA GCT CAG GCG TCC TAT GTA GAC GCG |
| 6A△C8-F | ATA GGACGC CTGAGC tgagaacttca |
| 6A△C8-R | GCT CAG GCG TCC TAT GTA GAC GCG CGG CAT |
| 6A△C9-F | TACATAGGACGCCTGtgagaacttcaggg |
| 6A△C9-R | CAG GCG TCC TAT GTA GAC GCG CGG CAT GTC |
| 6A△C9△N2-F | GGCATCTACGTCGAC CGCGTC TACATA |
| 6A△C9△N2-R | CAG GCG TCC TAT GTA GAC GCG GTC GAC GTA |

**Primers for generating T7 MS2-SRSF9 mutants**

| **Names** | **Sequence (5′--> 3′)** |
| --- | --- |
| 9 RRM1 salΙ-F | ACGTCGACGGGCGCATCTACGTGGGGAACCTTCCGACCGAC |
| 9 RRM1 BamhΙ-R | ACG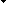GATC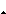C C TA AGT CCT GGG GAA CTC CAC ACG AAG C |
| SLIC 9 RRM2 -F | CAACTCCGGCATCTACgtcgacTTCCGAGTTCTTGTTTCAGGAC |
| SLIC 9 RRM2 -R | CCC AAA CTC ACC CTG AAG TTC TCA GCT TCT CTC AGG ATA AAC TC |
| SLIC 9 RS domain -F | CAACTCCGGCATCTACgtcgacACCAGCTATGGCTACTCACGGT |
| SLIC 9 RS domain -R | CCC AAA CTC ACC CTG AAG TTC TCA TCA GTA GGG CCT GAA AGG AGA |
| SLIC 9 RRM1/Gly-rich -F | CAACTCCGGCATCTACgtcgac GGGCGCATCTACGTGGGGAAC |
| SLIC 9 RRM1/Gly-rich -R | CCC AAA CTC ACC CTG AAG TTC TCA ATC AGA TCT TCT TGT AGG AG |
| SLIC 9 △RS domain -F | CAACTCCGGCATCTACgtcgac GGGCGCATCTACGTGGGGAAC |
| SLIC 9 △RS domain -R | CCC AAA CTC ACC CTG AAG TTC TCA GCT TCT CTC AGG ATA AAC TC |
| SLIC 9 Gly-rich/RRM2 -F | CAACTCCGGCATCTACgtcgacGGAGGTCGGGGTGGGTGG |
| SLIC 9 Gly-rich/RRM2 -R | CCC AAA CTC ACC CTG AAG TTC TCA GCT TCT CTC AGG ATA AAC TC |
| SLIC 9 △RRM1 -F | CAACTCCGGCATCTACgtcgacGGAGGTCGGGGTGGGTGG |
| SLIC 9 △RRM1 -R | CCC AAA CTC ACC CTG AAG TTC TCA TCA GTA GGG CCT GAA AGG AGA |
| SLIC 9 RRM2/RS domain -F | CAACTCCGGCATCTACgtcgacTTCCGAGTTCTTGTTTCAGGAC |
| SLIC 9 RRM2/RS domain -R | CCC AAA CTC ACC CTG AAG TTC TCA TCA GTA GGG CCT GAA AGG AGA |
| SLIC 9 RRM1△C9-F | CAACTCCGGCATCTACgtcgacGGGCGCATCTACGTGGGGAACCTTCCGACCGAC |
| SLIC 9 RRM1△C9-R | CCC AAA CTC ACC CTG AAG TTC TCA ACA CTG GCC ATA ATC ATA ACC |
| SLIC 9 RRM1+N-F | CAACTCCGGCATCTACgtcgacATGTCGGGCTGGGCGGACGAG |
| SLIC 9 RRM1+N-R | CCC AAA CTC ACC CTG AAG TTC TCA AGT CCT GGG GAA CTC CAC ACG A |
| SLIC 9 RRM1+N△C9-F | CAACTCCGGCATCTACgtcgacATGTCGGGCTGGGCGGACGAG |
| SLIC 9 RRM1+N△C9-R | CCC AAA CTC ACC CTG AAG TTC TCA ACA CTG GCC ATA ATC ATA ACC |
| 9 RRM1 E→R-F | TGTCGG CTT CGT GTGCGTTTC CCC AGGA |
| 9 RRM1 E→R-R | CAC ACG AAG CCG ACA CTG GCC ATA ATC ATA AC |
| 9 RRM1 ALL R-F | GATTATGGCCAGTGTCGG CGTCGTCGTCGTCGTCGTCGTCGTTAGGGATCC |
| 9 RRM1 ALL R-R | CCG ACA CTG GCC ATA ATC ATA ACC ATT TCT TCC A |
| 9 RRM1 R→E①-F | GATTATGGCCAGTGTGAGCTTCGTGTGG |
| 9 RRM1 R→E①-R | ACA CTG GCC ATA ATC ATA ACC ATT TCT TCC |
| 9 RRM1 R→E②-F | GGCCAGTGTGAGCTTGAGGTG GAG TTC C |
| 9 RRM1 R→E②-R | AAG CTC ACA CTG GCC ATA ATC ATA ACC ATT |
| 9 RRM1 R→E③-F | GAGGTGGAGTTCCCCGAGACTTAGGGATCC |
| 9 RRM1 R→E③-R | GGG GAA CTC CAC CTC AAG CTC ACA CTG GCC |
| 9 RRM1 ALL E-F | GTTATGATTATGGCCAGTGT GAGGAGGAGGAGGAGGAGGAGGAGGAGTAGGGATCC |
| 9 RRM1 ALL E-R | ACA CTG GCC ATA ATC ATA ACC ATT TCT TCC ATA AAT AGC A |
| 9 RRM1 ALL A-F | GTTATGATTATGGCCAGTGT GCTGCTGCTGCTGCTGCTGCTGCTGCTTAGGGATCC |
| 9 RRM1 ALL A-R | ACA CTG GCC ATA ATC ATA ACC ATT TCT TCC ATA AAT AGC A |
